# Modern brachytherapy in glioblastoma: overcoming clinical barriers to improve local control

**DOI:** 10.3389/fonc.2026.1867785

**Published:** 2026-07-20

**Authors:** Perrine Schneller, Joël Daouk, Julien Pierson, Fabien Rech, Muriel Barberi-Heyob

**Affiliations:** 1 Université de Lorraine, CNRS, CRAN, Nancy, France; 2 CHRU-Nancy, Service de Neurochirurgie, Nancy, France

**Keywords:** cancer therapy, dosimetry, glioblastoma, internal radiotherapy, Monte Carlo simulations, neuro-oncology, radiobiological effects, radionuclide

## Abstract

Glioblastoma almost invariably recurs at the margin of the resection cavity despite maximal treatment combining surgery, external beam radiotherapy, and chemotherapy. More than 80% of recurrences arise locally, underscoring a persistent unmet need for improved locoregional control. Despite major advances in conformal external beam radiotherapy techniques, durable local control remains challenging because of diffuse tumor infiltration, biological resistance, and the need to preserve surrounding healthy brain tissue. Although internal radiotherapy has existed for decades and is supported by a compelling radiobiological rationale, its integration into routine neuro-oncological practice remains limited. In this narrative review, we examine why a technically established approach, based on a sound biological rationale, continues to play a limited role in glioblastoma management. We focus on dosimetric standardization as a major challenge specific to internal radiotherapy. Unlike external beam radiotherapy, which prescribes absorbed dose within a standardized planning framework, internal radiotherapy delivers radioactive activity, resulting in spatially and temporally evolving dose distributions that are difficult to reconstruct, compare, and biologically interpret. Recent advances in intracavitary implantable and injectable platforms, patient-specific modeling, and improved understanding of glioblastoma radiobiology may enable a shift toward biologically informed treatment planning. By integrating radiation physics, tumor biology, and clinical decision-making, modern brachytherapy may emerge as a structured complement to multimodal glioblastoma care aimed at achieving improved local control.

## Highlights

- Modern brachytherapy directly targets the predominant site of glioblastoma recurrence
- Radionuclide radiobiology provides a mechanistic rationale for targeting resistant tumor niches
- New intracavitary platforms enable immediate post-resection irradiation within clinical workflows
- Standardized integrative dosimetry is critical for clinical adoption
- Brachytherapy provides short-range spatial dose intensification at the pericavitary interface

## 1 Clinical context of glioblastoma

Glioblastoma (GBM), defined in the current WHO 2021 classification as a diffuse astrocytic tumor, IDH-wildtype, is the most common and aggressive primary brain tumor in adults (1). The current standard-of-care for newly diagnosed GBM includes maximal safe surgical resection (2), followed by external beam radiotherapy (EBRT) combined with concomitant and maintenance temozolomide (TMZ) (3–5). In eligible patients, tumor-treating fields (TTFields), consisting of low-intensity, intermediate-frequency alternating electric fields delivered through transcranial arrays, may also be added during the maintenance phase. TTFields disrupt mitotic spindle organization and cytokinesis, thereby impairing tumor cell proliferation. This combination has been shown to improve progression-free and overall survival in selected patients (6). Although this multimodal approach modestly prolongs survival, long-term outcomes remain poor, with 5-year survival rates around 10% or less (4–7). A key limitation of current treatment is the high rate of local recurrence. Most patients relapse within months after standard treatment, and recurrence predominantly occurs locally, with more than 80% of recurrences reported at or near the resection cavity in several clinical series (7–9). However, recurrence patterns are dynamic and may evolve over time. Although early failures are predominantly local, distant or multifocal recurrences may occur, particularly in patients achieving durable local control after focal therapies (8–10). This spatial pattern of failure suggests that GBM progression is driven less by distant dissemination than by persistent microscopic disease in the pericavitary brain. Tumor growth kinetics in GBM must be interpreted carefully, as cellular doubling time and volumetric tumor doubling time are not equivalent. Macroscopic volumetric growth reflects not only tumor cell proliferation, but also cell death, necrosis, hypoxia, edema, vascular changes, and spatial constraints within the brain. Nevertheless, reported volumetric doubling times in untreated GBM are often on the order of a few weeks, supporting the clinical relevance of minimizing delays between surgery and radiotherapy initiation. For recurrent GBM, therapeutic options include re-resection (11, 12), re-irradiation (13–16), chemotherapy [*e.g.* enzastaurin, carmustine, lomustine, high-dose TMZ (17–21)], immunotherapy (22, 23), targeted agents (16–20, 23–26), or combinations thereof. However, none of these strategies has substantially altered overall prognosis, and median survival after recurrence remains limited. Given this predominant pattern of local failure, enhancing locoregional tumor control has become a key priority. GBM cells exhibit strong invasive potential into the peritumoral brain (27), which complicates complete resection and renders microscopic disease at the margin virtually unavoidable. Consequently, EBRT remains a cornerstone of treatment in newly diagnosed GBM and continues to play an important role in selected recurrent settings.

Several advanced EBRT modalities, fractionated stereotactic radiotherapy (27, 28), stereotactic radiosurgery (29–31), proton beam therapy (32), and boron neutron capture therapy (33, 34), have been explored to improve local control. Despite these advances, all remain constrained by normal tissue toxicity, radiobiological constraints related to dose fractionation, and limited efficacy against glioma stem-like cells (GSCs). Moreover, delay between surgery and EBRT initiation leaves a window during which residual tumor cells may proliferate unchecked, potentially driving early progression and shortening survival (35). Even after macroscopically complete resection followed by standard adjuvant EBRT and TMZ, the surgical margin remains the predominant site of tumor recurrence and progression (36, 37). This pattern reflects not only the biological aggressiveness of GBM but also the limited capacity of current imaging modalities, including MRI (magnetic resonance imaging), to detect and delineate microscopic infiltration beyond contrast-enhancing regions (38).

Biological resistance further complicates local control. GSCs, often concentrated in peritumoral niches, display enhanced DNA damage response and repair capacities through ATM/ATR (ataxia-telangiectasia mutated/ataxia-telangiectasia and Rad3-related) signaling pathways (39), thereby contributing to radioresistance. Hypoxic microenvironments and molecular heterogeneity further contribute to differential radiation sensitivity across the tumor bed. Together, these spatial and molecular factors explain why GBM preferentially recurs within 2 cm of the surgical cavity. However, this infiltrative radius extends beyond the effective dose range of most current brachytherapy approaches, raising important questions regarding its potential therapeutic impact. A key working hypothesis is that brachytherapy preferentially targets the region of highest residual tumor cell density at the resection margin, which is considered the primary driver of early local progression. In this framework, focal dose intensification at the pericavitary interface may delay or prevent early relapse, while more diffusely infiltrating tumor cells located beyond the high-dose region remain addressed by EBRT and systemic therapies.

To address these limitations, several strategies have been explored, including hypofractionated and simultaneous integrated boost techniques, which modestly improve local control (14, 40). These limitations have renewed interest in radiation approaches capable of delivering immediate and spatially confined dose intensification at the site of highest recurrence risk. Among these, modern brachytherapy techniques have re-emerged as particularly well suited to address aspects of the spatial and biological challenges of GBM. By enabling immediate irradiation of the tumor bed at or shortly after surgery, brachytherapy reduces the delay between resection and radiation delivery while limiting exposure to surrounding healthy brain tissue. Experimental approaches such as intraoperative radiotherapy (41) and convection-enhanced delivery (CED) of radionuclides (42) further illustrate this trend, although intracavitary brachytherapy remains the most clinically developed strategy. However, the persistent inability to prevent local recurrence after maximal therapy highlights the need for more precise and durable locoregional treatments. Nevertheless, improving targeting alone is insufficient if radiation dose cannot be accurately quantified, reconstructed, and biologically interpreted at the pericavitary interface. This limitation is particularly critical in internal radiotherapy, where dose deposition depends not only on geometry but also on radionuclide decay, tissue heterogeneity, and temporal activity distribution. Thus, beyond device design, a major translational challenge for internal radiotherapy lies in dosimetric control and biological dose modeling.

This narrative review addresses a central paradox in GBM treatment by examining persistent barriers to local control despite modern EBRT, the mechanistic rationale for perioperative internal radiotherapy, and integrative dosimetry as a key barrier to clinical translation.

## 2 Barriers to local control despite modern EBRT

EBRT remains the cornerstone of treatment for newly diagnosed GBM and has benefited from major advances in treatment planning, image guidance, and dose conformity through techniques such as IMRT, VMAT, stereotactic radiotherapy, and proton therapy. Despite these advances, durable local control remains difficult to achieve. This persistent challenge reflects a combination of biological resistance mechanisms, diffuse tumor infiltration beyond radiologically visible disease, and the need to preserve surrounding normal brain tissue. Consequently, several barriers continue to limit safe treatment intensification despite substantial improvements in radiation delivery. These barriers can be broadly categorized into three groups: (i) normal tissue toxicity, (ii) biological resistance mechanisms, and (iii) spatial limitations related to the diffuse infiltrative nature of GBM.

First, dose escalation remains constrained by the limited radiotolerance of healthy brain tissue. Brain radiotolerance is highly heterogeneous and depends on irradiated volume, fraction size, cumulative dose, prior irradiation, and anatomical location. Critical structures such as the brainstem, optic pathways, hippocampal regions, and eloquent white matter tracts exhibit limited tolerance to high cumulative or large per-fraction doses. Historical radiotolerance estimates should also be interpreted cautiously, as older studies were often based on less conformal irradiation techniques, less reliable dosimetric reconstruction, and, in some cases, lower-energy X-rays associated with different radiation quality, LET (linear energy transfer), and relative biological effectiveness. Treatment-related toxicities, including radiation necrosis and neurocognitive effects, remain important considerations when evaluating dose escalation and long-term functional outcomes, particularly in patients achieving prolonged disease control. Second, several biological features intrinsic to GBM compromise the effectiveness of EBRT. These include the radioresistance of GSCs, mediated by enhanced DNA repair capacity and localization to hypoxic niches (27), as well as diffuse tumor infiltration, especially along white matter tracts, which allows tumor cells to escape focal irradiation. Third, the high proliferation rate of GBM further limits the impact of conventional radiation schedules. In treatment-naive patients, the median volumetric doubling time has been estimated in some studies at ~21 days, corresponding to a daily growth rate of 2.1% (43, 44). This implies that significant tumor regrowth may occur during the six-week EBRT regimen, undermining its therapeutic effect. Delays in treatment initiation or interruptions during fractionation further exacerbate this issue (35, 45). Although strategies such as accelerated fractionation have been explored to counteract tumor repopulation, their clinical benefit remains limited due to increased toxicity.

In the following sections, we examine five major barriers to durable local control in GBM: radiation necrosis, cognitive decline, fractionation constraints, GSC radioresistance, and the persistence of migratory tumor cells, and discuss how modern brachytherapy may help address these challenges.

### 2.1 Radiation necrosis

Radiation necrosis is a recognized late complication of cranial irradiation, corresponding to a delayed injury of normal brain tissue that may occur months to years after treatment. Although potentially symptomatic and clinically significant, its incidence depends strongly on radiation dose, fractionation, irradiated volume, prior treatments, and diagnostic criteria used for its assessment. Reported incidences vary considerably across studies because of differences in treatment techniques, follow-up duration, imaging criteria, and patient populations. Higher cumulative doses, larger irradiated volumes, and elevated doses per fraction are generally associated with increased risk. While the incidence of radiation necrosis has decreased with contemporary conformal radiotherapy techniques and adherence to standard dose constraints, this complication remains an important consideration in the context of dose escalation, re-irradiation, and highly focal dose-intensification strategies. Mechanistically, radiation necrosis arises from radiation-induced endothelial damage, leading to BBB (blood-brain barrier) disruption (46–48). Higher cumulative doses and larger irradiated volumes are associated with increased risk, as brain endothelium is particularly radiosensitive. At the molecular level, ionizing radiation induces DNA double-strand breaks (DSBs) in endothelial cells, activating the ATM and ATR signaling pathways. ATM responds primarily to DSBs, triggering phosphorylation of downstream effectors such as p53, H2AX (γ-H2AX), and CHK2 (checkpoint kinase 2), leading to cell cycle arrest, repair, or apoptosis if damage is irreparable (49, 50). ATR is activated by replication stress or single-stranded DNA regions and promotes checkpoint activation through CHK (51). This endothelial damage is compounded by oxidative stress, including accumulation of reactive oxygen species (ROS), lipid peroxidation, and mitochondrial dysfunction, which further compromise BBB integrity (52). In parallel, pro-inflammatory cytokines such as TNF-α, IL-6, and IL-1β are released, activating NF-κB signaling and promoting immune cell infiltration (53).

Hypoxia plays an amplifying role in the development of necrosis. Microvascular injury and thrombosis impair oxygen delivery, further aggravating tissue damage. HIF-1α (hypoxia-inducible factor 1 alpha) is upregulated in response, leading to VEGF (vascular endothelial growth factor) expression and abnormal neoangiogenesis. However, these newly formed vessels are structurally immature and functionally inefficient, worsening ischemia rather than resolving it (54).

Additional risk factors include pre-existing tumor hypoxia, prior chemotherapy (e.g. TMZ), radiosensitizer use, and individual genetic susceptibility. Efforts to mitigate this toxicity have focused on reducing radiation exposure to healthy tissue, targeting oxidative stress and inflammation, or exploiting radioprotective agents. However, these approaches remain limited. Despite major advances in conformal radiotherapy techniques, treatment intensification remains constrained by the need to balance tumor control against the risk of injury to surrounding normal brain tissue. This limitation provides a rationale for exploring complementary local irradiation strategies, including modern brachytherapy approaches, which may further concentrate dose delivery within the pericavitary region while limiting exposure of adjacent brain structures.

### 2.2 Cognitive decline

Neurocognitive decline is a multifactorial complication in patients with GBM. The tumor itself contributes through infiltrative growth, tumor-associated necrosis, edema, seizures, neuroinflammation, and disruption of functional brain networks. Additional factors including surgery, systemic therapies, corticosteroid exposure, antiepileptic medication, disease progression, and treatment-related injury may further influence cognitive outcomes. In selected contexts, particularly when larger brain volumes or vulnerable regions are irradiated, EBRT may contribute to cognitive changes through radiation-induced effects on white matter tracts, vascular structures, and neurogenic niches, leading to deficits in memory, executive function, and processing speed (55). Periventricular and frontal white matter regions are particularly vulnerable, and injury in these areas may disrupt neural connectivity and contribute to neurocognitive deterioration. The severity of these effects has been associated with radiation dose and irradiated volume, although substantial inter-patient variability exists. Fractionation and modern conformal radiotherapy techniques mitigate, but do not entirely eliminate, this risk (47). In patients who achieve prolonged disease control, cognitive impairment may be cumulative and influenced by multiple interacting factors. Additional risk factors include advanced age, pre-existing neurological impairment, and individual susceptibility. Given the absence of curative treatment options for most GBM patients, preserving neurological function remains an important therapeutic objective. This creates a clinical challenge: treatment intensification may improve local tumor control but must be balanced against the risk of long-term neurotoxicity. Although modern techniques such as IMRT (intensity-modulated radiotherapy), VMAT (volumetric modulated arc therapy), proton therapy, and, when anatomically feasible, hippocampal-sparing approaches have substantially improved normal tissue preservation, long-term cognitive outcomes remain an important consideration in treatment planning. Research efforts now focus on neuroprotective strategies, including anti-inflammatory agents and therapies targeting glial reactivity or vascular injury. However, these remain largely experimental. By enabling highly localized radiation delivery, modern brachytherapy may offer a potential strategy to reduce exposure of critical neural structures while maintaining tumoricidal efficacy.

### 2.3 Fractionation constraints and dose limitations

EBRT for GBM typically follows a fractionated regimen of 60 Gy delivered in 30 fractions over six weeks, balancing efficacy and safety based on radiobiological principles. This standard schedule reflects a trade-off: maximizing tumor control while limiting damage to surrounding healthy brain.

The rationale for dose fractionation was established empirically long before the development of modern radiobiological models. Subsequently, the linear-quadratic (LQ) model provided a quantitative framework to describe and compare the biological effects of different fractionation schedules (56). In this model, biological effectiveness is expressed as E = αD + βD², where D is the delivered dose. Although the α and β parameters have often been associated with different components of radiation-induced cell killing, their precise biological interpretation remains debated. Nevertheless, the α/β ratio remains widely used in clinical radiobiology to compare tissue responses to fractionation. GBM is generally considered to have an α/β ratio around 10 Gy, whereas normal brain tissue exhibits a lower α/β ratio (approximately 2–3 Gy), supporting the rationale for conventional fractionation schedules that preferentially spare late-responding normal tissues. This differential response underpins the clinical rationale for fractionation but also contributes to its therapeutic limitations. In clinical practice, dose escalation beyond 70 Gy has not improved outcomes, mainly due to increased toxicity (57). Likewise, hyperfractionation, delivering smaller doses more than once daily, has not yielded significant survival benefits (58). Hypofractionated regimens (*e.g.* 40 Gy in 15 fractions) are sometimes used in elderly or frail patients to reduce treatment duration while maintaining acceptable efficacy (59). However, opportunities for further dose escalation remain constrained by normal brain tolerance and the limited therapeutic window available in GBM treatment. Overall, opportunities for safe dose intensification in GBM remain constrained by the narrow therapeutic window between tumor control and preservation of healthy brain tissue. These limits restrict our ability to intensify treatment at the tumor margin, precisely where residual infiltrative cells persist after surgery. These limitations provide a rationale for integrating complementary local irradiation strategies capable of intensifying treatment at the surgical margin without replacing EBRT.

### 2.4 Glioblastoma stem-like cells radioresistance

A major barrier to effective radiotherapy in GBM is the intrinsic radioresistance of GSCs. These cells represent a subpopulation within the tumor capable of self-renewal, differentiation, and tumor propagation and are particularly resilient to conventional treatments.

Radiation induces DSBs, a primary mechanism of cytotoxicity. However, GSCs exhibit enhanced DNA repair capacity, allowing them to rapidly recover from radiation-induced genomic damage. They overexpress key mediators of homologous recombination and DNA damage response, including RAD51 and other repair pathway components, which facilitate efficient repair and contribute to therapeutic evasion (39). Non-homologous end joining pathways, such as DNA-PK-mediated repair, also play a role in rapid but error-prone damage resolution. Importantly, GSCs localize preferentially to hypoxic niches, where oxygen deprivation further reduces radiation efficacy (60). This microenvironment not only protects existing GSCs but also promotes reprogramming of differentiated cells into a more stem-like phenotype. Within these niches, integrin α_v_β_8_–TGFβ1 signaling has been shown to maintain both GSC motility and radioresistance (61).

Clinically, the role of GSCs in recurrence is supported by the observation that GBM often recurs within the high-dose radiation field, suggesting that a subpopulation of highly resistant cells survives treatment (62). Single-cell transcriptomic analyses of recurrent tumors consistently reveal enrichment in stemness-associated gene signatures, reinforcing this concept (63). These findings have prompted the development of targeted strategies, including inhibitors of DNA repair enzymes (*e.g.* ATR or PARP inhibitors) and agents that modulate the hypoxic microenvironment (HIF inhibitors, oxygen mimetics) (64). However, their clinical impact remains limited to early-phase trials.

Because of their infiltration, plasticity, and intrinsic repair mechanisms, GSCs may not be completely eradicated by currently available local treatment modalities, including EBRT. Modern brachytherapy, particularly when using radionuclides emitting high-LET radiation such as alpha or Auger emitters, represents a biologically plausible but still investigational strategy that could contribute to targeting radioresistant GSC-enriched regions within the pericavitary zone.

### 2.5 Limited impact on migratory cells

Migratory GBM cells remain particularly challenging to eradicate with local treatment approaches, including EBRT, especially when they infiltrate along white matter tracts. This limitation is rooted in both the anatomical features of the brain and the adaptive biology of GBM cells. These tracts provide aligned axonal pathways and specific extracellular matrix components that facilitate directed tumor invasion. GBM cells exploit this permissive microenvironment through integrin-mediated adhesion, involving α_v_β_3_ and α_5_β_1_ integrins and downstream activation of FAK (focal adhesion kinase) (65). Key adhesion molecules, such as L1CAM (L1 cell adhesion molecule) and NCAM (neural cell adhesion molecule), further promote anchoring to axonal and glial structures. L1CAM also enhances migration *via* integrin crosstalk and exosome signaling (66). Although this microenvironment is generally inhibitory to cellular migration due to myelin-associated proteins such as Nogo-A, GBM cells circumvent this blockade. They secrete SPARC (secreted protein acidic and rich in cysteine), a matricellular protein that binds to Nogo-A and neutralizes its anti-migratory effect (67), demonstrating remarkable plasticity in their invasive behavior. In addition to structural cues, the biochemical and immunological context of these regions supports radioresistance. These regions are relatively hypovascular, promoting local hypoxia which further drives stemness and treatment resistance. Moreover, the immune microenvironment of infiltrated white matter may further contribute to tumor persistence and therapeutic resistance (68).

GBM cells migrating along white matter tracts frequently exhibit stemness-associated transcriptional programs involving factors such as SOX2 and OLIG2, which have been implicated in tumor persistence, treatment resistance, and recurrence (69). Emerging data also suggest that neuronal activity within these regions may enhance tumor progression, as GBM cells establish synapse-like interactions with neurons, activating survival pathways (70, 71).

In some preclinical models, irradiation has been associated with increased migratory behavior, including through activation of kinases such as MRCK (myotonic dystrophy kinase-related CDC42-binding kinase) (72). These findings suggest that the biological response of GBM cells to irradiation may influence migratory behavior, highlighting the complexity of achieving durable local control in this disease.

In this context, modern brachytherapy may offer a complementary therapeutic opportunity. By allowing direct, localized radiation delivery within the resection cavity and adjacent infiltrated areas, it may improve targeting of residual tumor cells at the resection margin, where tumor cell density remains highest. Experimental approaches such as CED further illustrate the interest in highly localized treatment strategies, but intracavitary brachytherapy remains the most clinically established embodiment of this concept.

## 3 Rational therapeutic advantages of internal radiotherapy

Despite major advances in modern EBRT, durable local control remains difficult to achieve in GBM. This persistent challenge provides a compelling rationale for investigating complementary locoregional treatment strategies, including internal radiotherapy. These modalities should be considered spatially and temporally complementary rather than competing. Brachytherapy enables early, localized irradiation immediately after resection, thereby reducing the post-surgical proliferative window during which residual tumor cells may expand prior to EBRT initiation. By delivering focal dose intensification at the pericavitary interface, it may increase the biologically effective dose in the region most critical for early tumor regrowth. Its spatial selectivity confines high-dose irradiation to the resection margin, limiting irradiation outside the immediate pericavitary region. Whether this translates into reduced rates of radiation necrosis or neurocognitive toxicity remains to be established in prospective clinical studies. In addition, continuous low-dose-rate irradiation, particularly when combined with high-LET radionuclides, may improve targeting of treatment-resistant tumor subpopulations, including hypoxic cells and GSCs.

**Figure 1:**
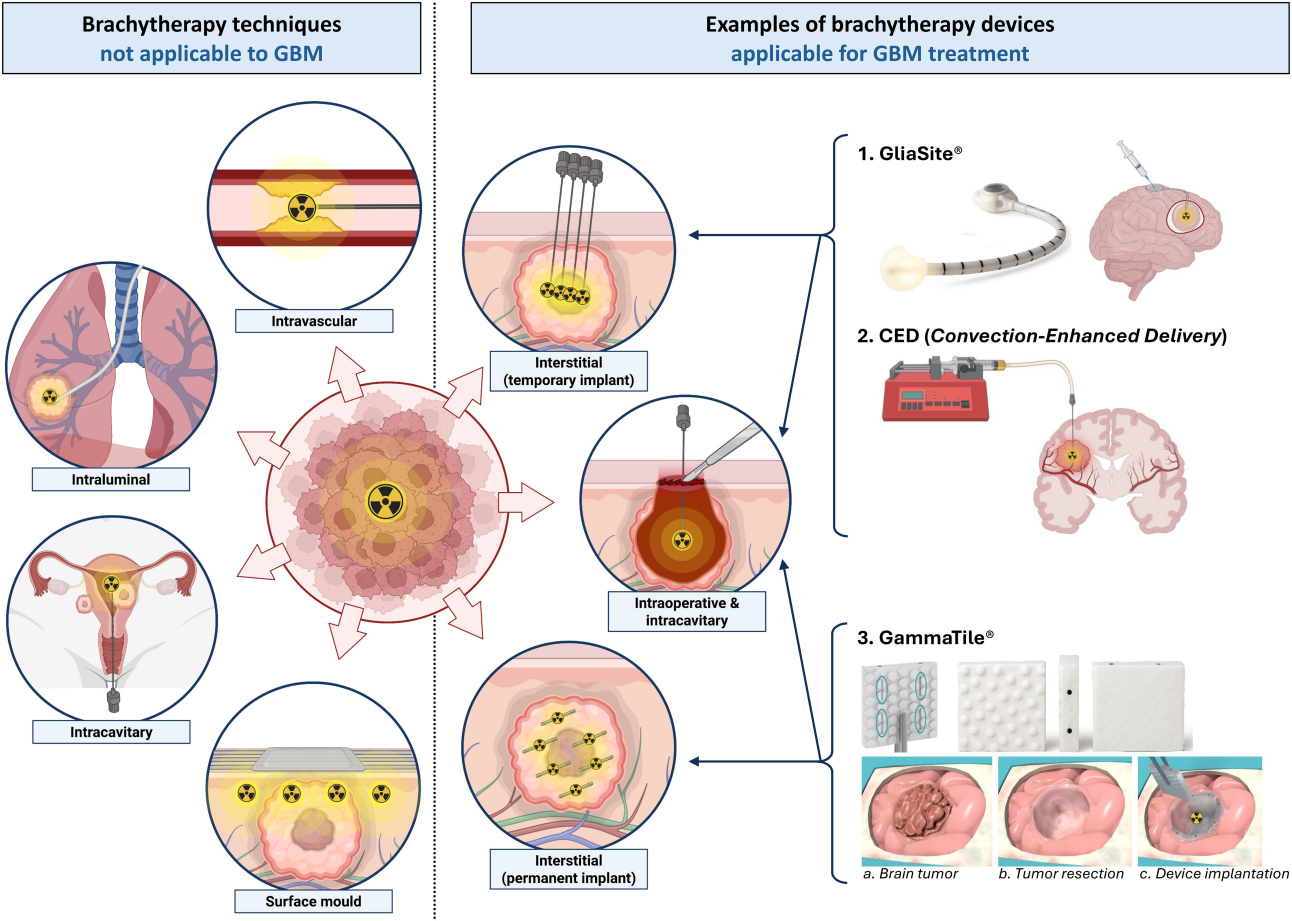
Interstitial brachytherapy involves placing radioactive sources directly into tumor tissue, delivering high doses of radiation to solid organs or soft tissues while minimizing damage to surrounding healthy areas. Intracavitary brachytherapy is used for tumors within body cavities, such as the uterus or cervix, with radioactive sources placed near the tumor for targeted radiation. Intraoperative brachytherapy delivers radiation during surgery, immediately after tumor removal, to enhance treatment effectiveness, especially for tumors in challenging locations such as GBM. Intraluminal brachytherapy targets tumors in hollow organs, such as the esophagus or bronchi, by placing radioactive sources inside the lumen. Intravascular brachytherapy treats vascular tumors or arterial blockages by delivering radiation directly into blood vessels. Finally, surface mould brachytherapy applies radiation to surface or near-surface tumors, often used for skin cancers or melanoma. Different devices and localized irradiation strategies have been developed for the treatment of GBM. (1) GliaSite^®^ is an expandable silicone balloon implanted immediately into the post-surgical cavity after tumor resection. In the case of brain tumors, the balloon is filled with a liquid ^125^I *via* an injection port on the surface of the skull (75). (2) Convection-enhanced delivery (CED) is a locoregional drug delivery technique that enables direct administration of therapeutic agents into the tumor while bypassing the BBB. Therapeutic compounds are infused through stereotactically implanted catheters using continuous low hydrostatic pressure generated by an infusion pump (79). Although not a brachytherapy technique *per se*, CED illustrates the broader interest in highly localized treatment strategies for GBM. (3) GammaTile^®^ is a flexible, bioresorbable collagen matrix incorporating titanium capsules containing ¹³¹Cs. Implanted directly on the edges of the excision cavity intraoperatively, this system enables immediate, localized irradiation (74). Created in BioRender. Schneller, P. (2026) https://BioRender.com/0f50who.

Accordingly, brachytherapy should not be viewed as an alternative to EBRT, but as a spatiotemporal complement that bridges the post-surgical interval and may reinforce early local tumor control. However, its therapeutic efficacy critically depends on accurate characterization and control of dose distribution within the pericavitary space. Modern brachytherapy, also referred to as interventional radiotherapy, has regained interest in GBM management in response to the persistent challenge of local recurrence. Advances in image-guided planning, precision delivery, and biocompatible carriers have enabled the development of clinically viable intracavitary systems. For example, GammaTile^®^ (73, 74) integrates cesium-131 (¹³¹Cs) seeds within a bioresorbable matrix for intraoperative implantation, while earlier platforms such as GliaSite^®^ (75–77) established key principles of intracavitary irradiation despite limitations related to toxicity. Although multiple brachytherapy approaches exist, including intracavitary, interstitial, intraoperative, surface, and intraluminal techniques (78) (**Figure 1**), only a subset is applicable to brain tumors. Early clinical studies have demonstrated feasibility and safety, but long-term comparative efficacy data remain limited, underscoring the need for structured clinical evaluation. Because of the limited range of most brachytherapy approaches, local irradiation alone is unlikely to eradicate the entire infiltrative compartment of GBM. Future therapeutic strategies will therefore likely require integration with systemic, molecularly targeted, immunomodulatory, or radiosensitizing approaches capable of addressing tumor cells beyond the high-dose pericavitary region.

### 3.1 Technological advances

Brachytherapy enables localized internal radiotherapy through direct placement of radioactive sources at the tumor site. In GBM, interstitial techniques insert isotopes into tumor tissue *via* catheters or needles (80–82), while intraoperative brachytherapy places sources immediately into the resection cavity during surgery, when tumor burden is minimal (73–77). This temporal proximity between resection and irradiation reduces the proliferative window that typically occurs before EBRT initiation.

Several brachytherapy modalities commonly used in other anatomical sites, including intraluminal, intravascular, and surface mould techniques, are generally not applicable to GBM because of the specific anatomical constraints of the brain (83–86). Among the evaluated approaches, intraoperative brachytherapy currently appears the most promising due to its immediate, localized irradiation and straightforward integration into the neurosurgical workflow. Platforms such as GammaTile^®^ are being evaluated in prospective trials (41). From a translational perspective, the key question is not only feasibility, but whether these platforms allow reproducible, quantifiable, and biologically interpretable dose delivery, a prerequisite for broader clinical adoption.

### 3.2 Selection of an appropriate radionuclide

Within this framework of early postoperative irradiation, radionuclide selection must be aligned with the spatial and biological characteristics of the pericavitary tumor microenvironment. Radionuclide selection should therefore be viewed as a therapeutic design parameter aligned with tumor biology and cavity geometry, rather than a purely physical property. Brachytherapy relies on α-particles, Auger electrons (AE), β^−^-particles, and γ-photons, each with distinct physical and biological properties. These classes differ in spatial range and radiobiological effects, which must be matched to tumor geometry and delivery constraints for optimal efficacy (**Table 1**). Beyond absorbed dose alone, the biological effectiveness of internal radiotherapy is strongly influenced by radiation quality. LET describes the amount of energy deposited per unit track length, whereas RBE reflects the biological effect produced relative to a reference radiation, typically megavoltage photons. In general, higher LET radiation produces denser ionization tracks, more complex DNA and chromosomal damage, and higher RBE values. For a given particle type, lower particle energies are generally associated with higher LET values. Consequently, physical dose alone may not adequately predict biological response, particularly when comparing radionuclides with markedly different radiation qualities. Brachytherapy enables highly localized dose deposition, with steep dose gradients that may help limit irradiation of nearby critical structures depending on source geometry and treatment planning (87). In addition to radionuclide emission characteristics, dose delivery mode also plays a critical role in therapeutic outcome. Low-dose-rate (LDR) brachytherapy provides continuous irradiation over extended periods, promoting sustained DNA damage, cell-cycle redistribution, and potential reoxygenation effects. In contrast, high-dose-rate (HDR) approaches deliver radiation in shorter, higher-intensity exposures, which may enhance direct cytotoxicity but offer less opportunity for biological modulation over time. These differences should be considered when optimizing treatment strategies in GBM, particularly in hypoxic and stem-like tumor cell populations. Although HDR and LDR brachytherapy may achieve similar physical dose distributions, their radiobiological effects can differ substantially. LDR irradiation delivers dose continuously over extended periods, allowing concurrent repair, redistribution, and reoxygenation processes, and may therefore be particularly relevant for targeting hypoxic tumor subpopulations. For radionuclides delivering dose over prolonged periods, biological effectiveness may be more appropriately described using dose-rate dependent radiobiological models incorporating repair kinetics, such as the generalized BED formalism proposed by Dale and colleagues (88). By contrast, HDR treatments generally require fractionation to preserve normal tissue tolerance, while practical limitations may arise from the duration of catheter implantation.

**Table 1:** Physical and radiobiological characteristics of α-, β^−^-, γ- and AE-emitters used in internal radiotherapy.

	Gamma (γ)	Beta^-^ (β^-^)	Alpha (α)	Electron Auger (AE)
LET and ionization pattern	Low (dependent on secondary electron LET) 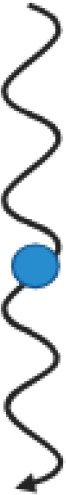	Low LET From 1 to 10 keV/µm^*^ ROS-mediated indirect damage 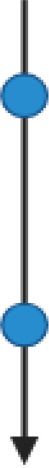	High LET From 50 to 230 keV/µm^*^ Direct, dense DNA ionization 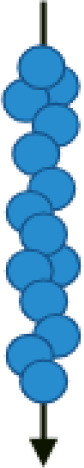	Medium/High LET From 4 to 26 keV/µm^*^ Direct, dense and very localized DNA ionization 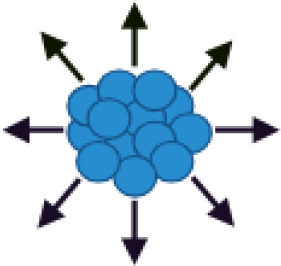
Penetration depth	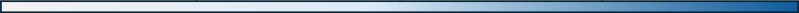			
	Deep penetration (cm); useful for imaging & brachytherapy 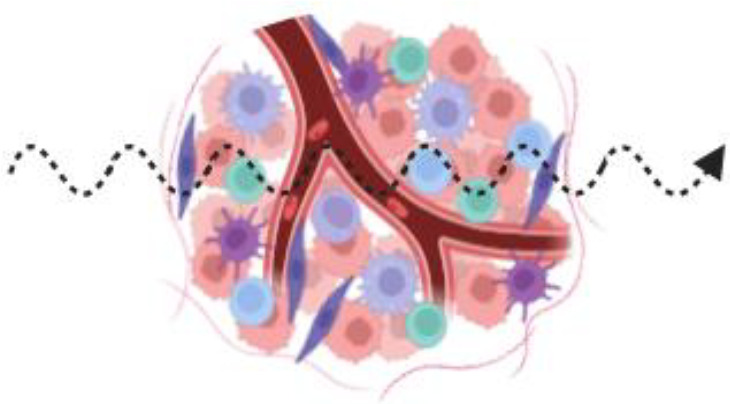	Intermediate (mm-cm); suitable for larger tumors 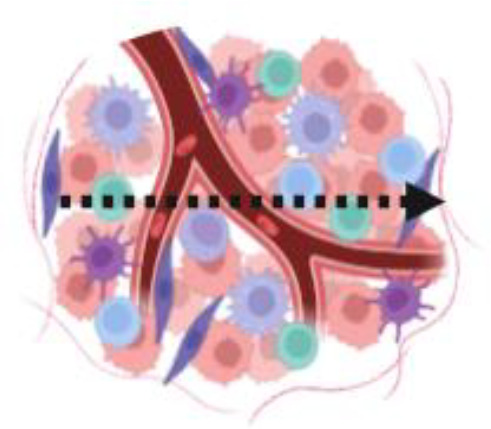	Short (40-100 µm); cell-level reach 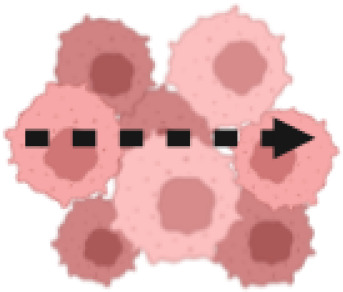	Very short (<100 nm); single-cell reach 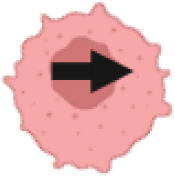
Healthy tissue impact	Requires shielding; deeper tissue exposure 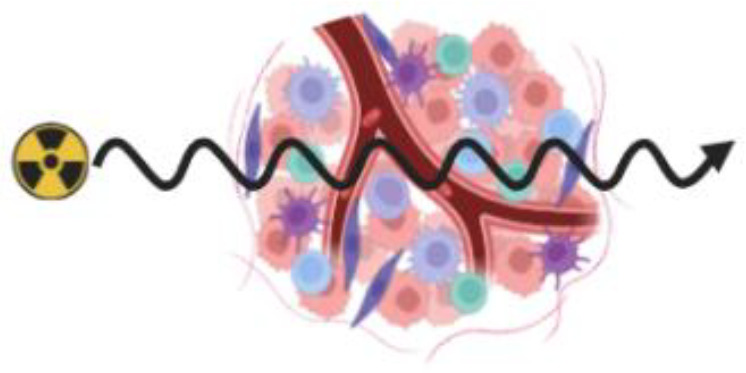	Broader dose deposition depending on range and targeting 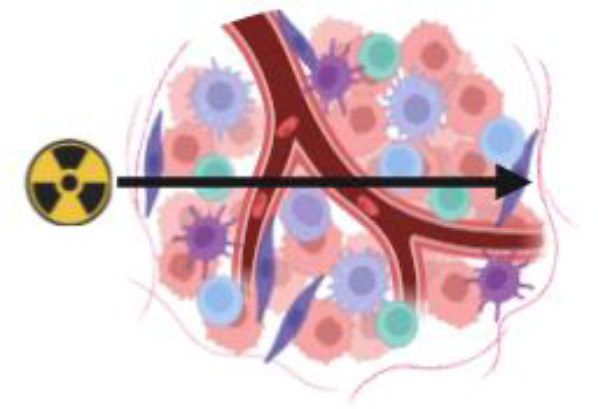	Highly localized dose deposition 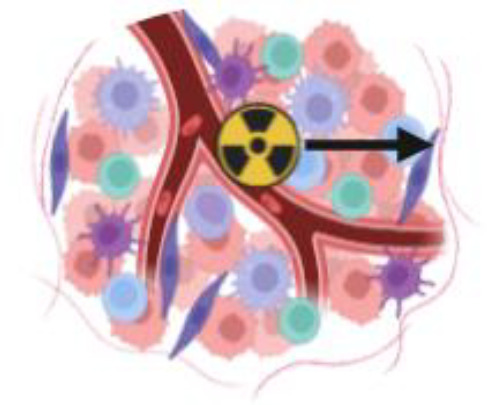	Hyper-localized; with targeting, limited effect outside targeted cells 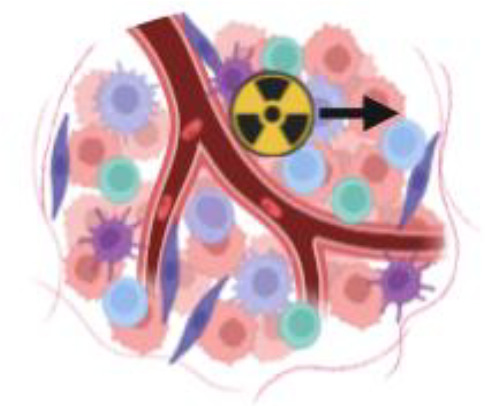
DNA damage	Indirect damage *via* secondary ionization 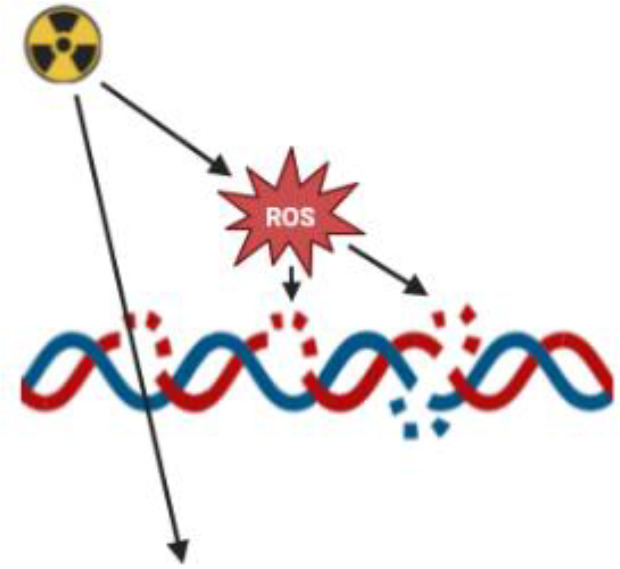	Mostly SSBs; fewer DSBs; repairable 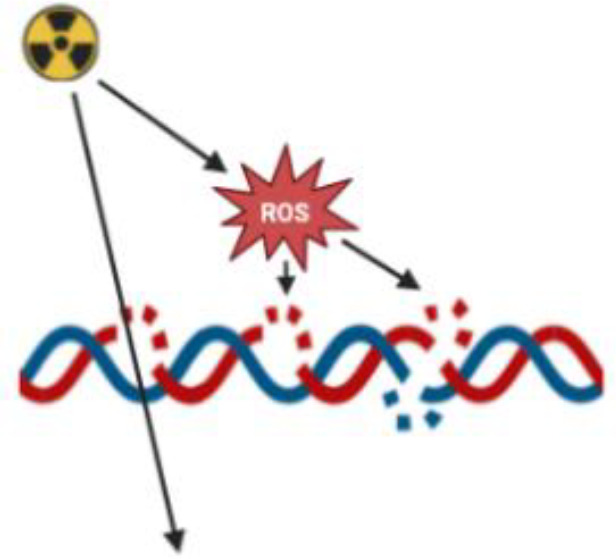	Clustered DSBs; complex lesions → apoptosis 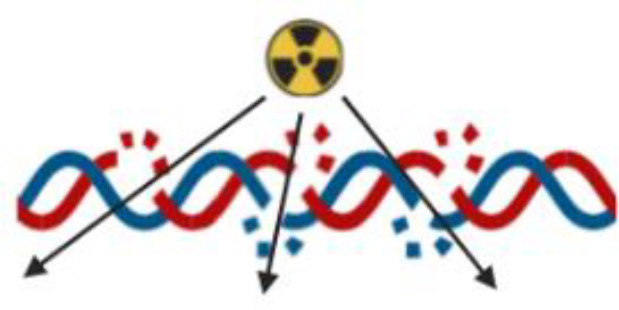	Ultra-localized and clustered DSBs; complex lesions → apoptosis 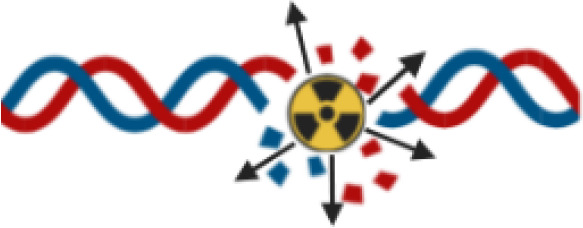
Hypoxia sensitivity	Poor performance in hypoxic environments	Reduced efficacy (ROS require oxygen)	Effective in hypoxia (oxygen-independent action)	Effective in hypoxia (oxygen-independent action)
Applications	Deep-seated tumors; PET/SPECT; external radiotherapy	Larger/diffuse tumors; often targeted	Micro metastases, small solid or hematologic tumors	Tumors expressing a specific target

This table summarizes key differences in LET, penetration depth, DNA damage pattern, and biological performance under hypoxia. α-particles and AE exhibit high LET and induce clustered, difficult-to-repair DNA damage. β^−^- and γ-emissions produce more diffuse and ROS-dependent effects. α- and AE-emitters have the potential to be effective in resistant tumor niches due to their hypoxia-independent modes of action. AE-emitters require precise nuclear localization for maximal efficacy, whereas α-emitters primarily rely on close proximity to target cells.

*Linear energy transfer (LET) is the amount of energy deposited by ionizing radiation per unit path length as it traverses matter, typically expressed in keV/μm. Low-LET radiation deposits energy sparsely along its track, whereas high-LET radiation produces dense ionization clusters. The reported LET ranges are representative values and may vary depending on the irradiated medium and particle energy. For a given particle type, LET generally decreases with increasing particle energy.

Created in BioRender. Schneller, P. (2026) https://BioRender.com/v952wa1.

Bold text is used for emphasis only to highlight the key features of each radiation type.

Alpha-particle emitters deliver high-LET radiation that induces complex, difficult-to-repair DNA damage within a range of 50-100 µm (89). Their very short range may be advantageous for targeting residual tumor cells located immediately adjacent to the resection cavity while minimizing irradiation of surrounding normal tissue. However, this limited penetration also represents a challenge in GBM, where infiltrative tumor cells may extend several millimeters to centimeters beyond the surgical margin. Because α-induced DNA damage is less dependent on oxygen, these emitters may retain efficacy in hypoxic niches that limit conventional photon-based RT. DaRT, based on ²²^4^Ra seeds releasing ²²^0^Rn, demonstrated slowed tumor growth in U87 xenografts and feasibility in a swine brain model (90, 91). Although promising, α-emitter-based intracranial brachytherapy remains investigational and is not part of routine clinical practice in GBM. While α-emitters require close proximity to target cells, achieved in other contexts through antibodies or nanoparticles (92–95), intracavitary brachytherapy may inherently facilitate such proximity at the resection margin. Daughter recoil remains a consideration (96). Nevertheless, their short effective range imposes stringent requirements for accurate spatial dosimetric modeling to avoid geographic miss at the cavity margin.

Radionuclides emitting AE are characterized by sub-100 nm range and LET of 4–26 keV/µm, requiring emission in immediate proximity to DNA for maximal effect (79, 97). This has been demonstrated using nuclear-targeting vectors in GBM models. ¹²³I-MAPi increased γ-H2AX foci and extended survival in TS543 PDX models (98), and [¹²^5^I]-UdR reduced GBM cell survival and migration with additive TMZ benefit (99). These findings highlight that AE efficacy is highly dependent on subcellular localization, making pharmacokinetics and intracellular trafficking critical determinants of therapeutic success. Short-range AE emissions from isotopes such as ¹²^5^I may contribute to cytotoxicity at the cavity interface. However, clinical translation requires integrating molecular targeting efficiency into dosimetric calculations, which remains rarely achieved in current practice.

Emitters of β^−^ have lower LET, 2–15 keV/µm, and millimetric penetration (100–103), enabling irradiation beyond the cavity wall to target infiltrative tumor cells. Their physical range more closely matches the 1–2 cm infiltrative margin characteristic of GBM recurrence. This principle is illustrated by vectorized β^−^ approaches: LNC-¹^88^Re increased survival in rat GBM models (104), CXCR4 targeting enhanced uptake and efficacy (105), and ^177^Lu-AuNPs achieved long-term survival in U-251 models (106). Although based on CED or nanocarriers, these results reflect how β^−^ range matches the infiltrative margin around the resection cavity. Clinically, β^−^ emitters may offer a compromise between spatial reach and safety, but require careful dose reconstruction to prevent excess irradiation of adjacent white matter.

The emission of γ-photons results in substantial deep penetration and their KERMA (kinetic energy released per unit mass) depends strongly on photon energy. Because photons are indirectly ionizing radiation, LET is generally considered through the secondary electrons generated during photon interactions (100). Accordingly, γ-rays are generally considered low-LET radiation because the secondary electrons generated during photon interactions exhibit relatively low LET values and RBE values close to unity. Their role in brachytherapy is central to dosimetry, imaging, and controlled dose gradients (107–110). Clinically, ¹²^5^I (GliaSite^®^) and ¹³¹Cs (GammaTile^®^) emit low-energy photons (30–35 keV) that create steep dose fall-off from the cavity wall while enabling imaging and treatment planning (73–77, 111). In contrast to vectorized radionuclide approaches, stable intracavitary seed platforms facilitate post-implant dosimetric reconstruction, a key requirement for regulatory and clinical standardization. However, translating these properties into clinical benefit requires standardized dosimetric modeling and patient selection criteria.

## 4 Impact of radionuclide choice and delivery geometry on dose distribution

**Figure 2:**
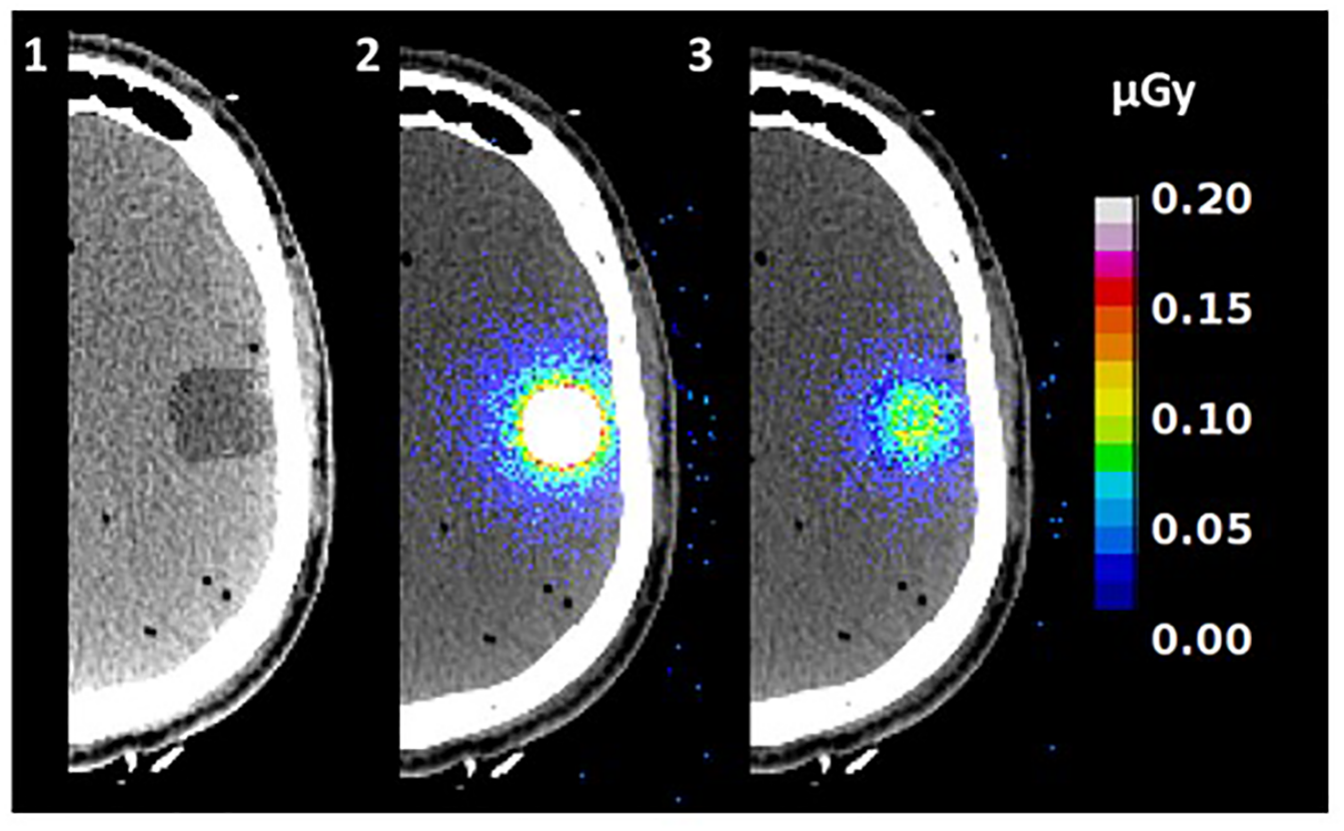
Simulated dose maps, following GBM resection. (1) CT scan used for simulation; (2) dose distribution from Gliasite^®^, defined by balloon inflation; (3) GammaTile^®^ distribution, showing heterogeneity around individual seeds and lower diffusion outside the implant zone. Simulations were performed using GATE v9.3 for a normalized activity (1 MBq), to illustrate relative dose distributions.

Dose escalation in intracavitary brachytherapy depends not only on radionuclide emission properties, but also on the geometry of the delivery platform. This is illustrated in **Figure 2**, which compares simulated dose maps in a post-resection GBM cavity for liquid ¹²^5^I balloon brachytherapy and solid ¹³¹Cs seed-based brachytherapy.

In the GliaSite^®^ system, the liquid ¹²^5^I source conforms to the inflated balloon, generating a spherical and relatively homogeneous dose distribution from the cavity wall. Low-energy photons (28–35 keV), together with associated short-range Auger electron emissions, result in dose deposition primarily confined to a few millimeters from the cavity interface (75, 111). In contrast, GammaTile^®^ relies on discrete ¹³¹Cs seeds embedded in a collagen matrix. This architecture produces a heterogeneous pericavitary dose distribution, characterized by localized gradients around each seed and reduced diffusion outside the implant zone (74). Although both isotopes emit photons of similar energy, the solid seed geometry fundamentally alters dose conformity. This comparison demonstrates that, in intracavitary brachytherapy for GBM, device architecture is as critical as radionuclide selection in shaping dose distribution at the pericavitary interface.

Emerging injectable platforms such as ¹^66^Ho-chitosan hydrogels represent a third paradigm, where the radionuclide source conforms entirely to the three-dimensional geometry of the cavity. Preclinical studies suggest that such hydrogel-based approaches may offer highly conformal cavity coverage while providing opportunities for adaptable dosimetry (112), potentially addressing some dosimetric limitations observed with both balloon-based and seed-based systems. This approach further highlights how adaptable delivery geometry may serve as a key lever for improving dosimetric control in GBM.

## 5 Internal radiotherapy strategic optimizations

Despite growing clinical and preclinical evidence, internal radiotherapy remains underutilized in GBM treatment. As discussed above, this limitation is not primarily due to technical feasibility, but rather to the absence of standardized dosimetric and radiobiological frameworks equivalent to those established for EBRT. In EBRT, dose delivery is planned, calculated, and prescribed in Grays (Gy) using well-defined beam geometry, tissue models, and fractionation principles. By contrast, internal radiotherapy relies on the implantation or administration of a fixed radioactive activity (MBq or GBq), and the resulting dose distribution evolves dynamically over time as a function of radionuclide decay, spatial diffusion, biological clearance, and post-surgical anatomical changes. This paradigm shift makes dose prediction, optimization, and inter-patient comparison particularly complex. As a result, variability in patient selection, cavity geometry, radionuclide choice, and dose optimization persists across institutions, limiting reproducibility and hindering integration into clinical guidelines.

Operational constraints further contribute but remain secondary to this core limitation. Internal radiotherapy requires tight coordination between neurosurgery, radiation oncology, and nuclear medicine, and its deployment remains challenging outside academic centers with access to radiopharmaceutical infrastructure. From an interventional perspective, intracranial brachytherapy procedures are inherently complex and require multidisciplinary coordination. This procedural complexity has recently been formalized through the COMIRI (COMplexity Index of Interventional Radiotherapy Implants), which classifies brachytherapy interventions based on procedural, technical, and organizational parameters, and may contribute to standardizing implementation across centers. Nevertheless, these logistical barriers are secondary to a more fundamental issue: the difficulty in accurately predicting and controlling the spatial and biological effects of internally delivered radionuclides in the complex post-resection GBM environment.

### 5.1 The role of integrative dosimetry

Addressing this limitation requires moving from empirical activity-based delivery toward predictive, patient-specific dosimetry. In this context, Monte Carlo (MC) simulations have become essential tools for advancing internal radiotherapy toward reliable treatment planning. Unlike conventional dosimetry approaches based on simplified assumptions of tissue homogeneity or static geometry, MC methods enable patient-specific modeling of radiation transport within irregular post-resection cavities and surrounding infiltrated regions.

Conventional TG-43 formalism, the standard water-based dosimetric model recommended by the American Association of Physicists in Medicine (AAPM) and widely used in clinical brachytherapy, assumes homogeneous media and neglects tissue heterogeneities, which may limit its accuracy in the complex post-resection brain environment. More advanced model-based approaches, such as TG-186 recommendations, enable a more realistic representation of radiation transport by accounting for tissue composition, cavity geometry, and material interfaces. Monte Carlo simulations further extend these approaches by allowing fully patient-specific and dynamic modeling of dose deposition.

This precision is particularly relevant in GBM, where recurrence almost systematically originates within millimeters of the surgical margin. MC simulations can integrate fine-grained variations in tissue composition, including necrotic zones, infiltrated parenchyma, and radiosensitive subregions, allowing dose distribution to be tailored to biologically relevant high-risk areas while minimizing exposure to healthy brain. Importantly, MC modeling allows discrimination between the very different spatial behaviors of α-particles, β^−^-particles, AE, and γ-photons used in internal radiotherapy. High-LET α-particles require micrometric spatial accuracy, whereas β^−^- and γ-emissions require modeling over larger volumes to balance therapeutic reach and tissue sparing. Capturing these differences is essential for defining meaningful therapeutic windows.

The intraoperative setting adds another level of complexity. Post-resection cavity deformation, tissue relaxation, and hemorrhagic residues can significantly modify dose distribution. MC simulations provide a unique advantage by allowing recalibration of dosimetric models in response to these dynamic anatomical changes. This enables a shift from empirical implantation toward quantifiable and reproducible dose prescriptions.

### 5.2 From physical dose to biological effect

Recent MC platforms extend beyond physics by integrating biological endpoints such as DNA damage and cell survival. The development of Geant4-DNA has enabled nanometric-scale simulations relevant to short-range emitters such as α-particles and AE (113).

These models simulate track structure, DSB induction, and ROS diffusion in space and time. Although many validation studies rely on reference cell lines such as HSG (114), recent work has begun distinguishing between SSBs and DSBs (115, 116) and modeling ROS evolution following irradiation (117). However, these advances also highlight a critical gap between physical dose modeling and biological outcome prediction. This multiscale approach is particularly important for internal radiotherapy, where the biological effect is not solely determined by absorbed dose but by how energy is deposited at the subcellular level in heterogeneous tumor environments. Such multiscale modeling is essential if internal radiotherapy is to reach the level of biological predictability expected in modern radiation oncology.

### 5.3 The missing link: glioblastoma-specific radiobiology

A major limitation in current internal radiotherapy optimization is the insufficient characterization of how different radionuclides affect GBM cells and surrounding brain tissue. While the physical properties of α-, AE-, β^−^-, and γ-emitters are well described, their biological effects in GBM models remain incompletely understood. The response of glioma stem-like cells, hypoxic niches, infiltrative margins, and adjacent white matter to these emissions is still poorly documented (118–120). Most preclinical evaluations focus on cytotoxicity, but rarely assess effects on DNA repair pathways, cell migration, invasion capacity, or interactions with the tumor microenvironment. Likewise, the impact on normal brain cells, including neurons, astrocytes, and oligodendrocytes, remains insufficiently characterized in pericavitary contexts. The radiobiological consequences of radiation exposure beyond the immediate pericavitary region also remain poorly characterized. While dose distributions from brachytherapy decrease rapidly with distance, larger volumes of normal brain may still receive low-to-intermediate doses that may affect neuroinflammation, white matter integrity, vascular function, and long-term cognitive outcomes (46–48). Defining biologically relevant dose thresholds for these normal tissue compartments remains an important challenge for treatment optimization.

Without such data, translating MC-based physical simulations into biologically meaningful, patient-specific treatment planning remains limited. Dose reconstruction therefore remains disconnected from clinically relevant endpoints such as progression-free survival or neurotoxicity. Reported α/β estimates for GBM generally range between 8 and 10 Gy, consistent with a relatively early-responding tumor phenotype, although substantial uncertainty remains due to methodological differences and biological heterogeneity across studies. In this context, although BED modelling provides a useful framework for comparing fractionation schedules and integrating external beam radiotherapy with brachytherapy, its interpretation in GBM should remain cautious because of the biological heterogeneity of the disease, uncertainties surrounding the α/β ratio, and the limitations of current radiobiological models.

Beyond these modelling limitations, important biological uncertainties also remain regarding the behavior of radioresistant GBM subpopulations, particularly within hypoxic niches. Hypoxia is not only a modifier of radiosensitivity, but also a key regulator of GSC phenotype and tumor growth kinetics. Experimental studies have shown that hypoxic niches can maintain stem-like properties through HIF-dependent pathways, particularly HIF-2α, while promoting quiescent or slowly proliferating states (121, 122). Consequently, cellular proliferation dynamics and volumetric tumor growth may become partially dissociated. Importantly, quiescent GSC populations may escape therapies targeting proliferating cells and later re-enter the proliferative pool, contributing to tumor repopulation and recurrence (123). However, the duration over which hypoxic GSCs can persist, retain stemness, and resume proliferation remains poorly defined and represents an important area for future radiobiological investigation.

### 5.4 Toward biologically informed internal radiotherapy planning

To move beyond empirical radionuclide implantation, internal radiotherapy strategies must integrate: patient-specific MC dosimetry, radionuclide-specific spatial modeling, and GBM-specific radiobiological response data. Only through this integration can this approach achieve the same level of planning reliability as EBRT currently provides.

Such an approach would enable rational vector design, optimized radionuclide selection, improved implantation timing, and safer definition of therapeutic windows. Bridging this gap represents the central challenge for the clinical translation of this strategy in GBM. Ultimately, this framework is essential for transforming this approach from a technically feasible option into a standardized, biologically informed therapeutic strategy in neuro-oncology. Prospective validation in biomarker-stratified cohorts will be necessary to establish its clinical value.

## 6 Strategic outlook: integrating internal radiotherapy into the evolving landscape of glioblastoma management

To move beyond a descriptive account of existing technologies, we propose a forward-looking comparative framework for radiation delivery strategies in GBM, aimed at clarifying their respective positions along the translational continuum. Each modality, EBRT, IORT, CED, and internal radiotherapy, presents a distinct profile in terms of spatial precision, dosimetric control, and clinical maturity. EBRT remains the cornerstone of GBM treatment and provides highly conformal dose delivery. Nevertheless, durable local control remains challenging because of diffuse tumor infiltration, biological heterogeneity, and the need to preserve normal brain tissue. IORT addresses the temporal gap immediately post-resection but lacks adaptability to the evolving geometry of the surgical cavity and delivers a single, non-modulable dose. CED bypasses the BBB and improves local distribution of therapeutic agents, yet its technical complexity and limited diffusion radius can impair homogeneous tumor bed coverage. In contrast, internal radiotherapy enables early, sustained, and localized irradiation with intrinsic spatial selectivity. When combined with advanced delivery systems such as nanovectors, hydrogels, or bioabsorbable matrices, this approach becomes particularly well positioned to address radioresistant and selected infiltrative tumor compartments within the post-surgical microenvironment.

This comparative analysis supports a rationale for integration rather than substitution. Internal radiotherapy appears particularly well adapted to the post-resection context, where residual stem-like and migratory cells persist despite maximal surgery and systemic therapy. Accordingly, it should be positioned as a complementary component within multimodal care pathways rather than a competing modality. Rather than replacing EBRT or systemic approaches, it may function as a spatiotemporal bridge, filling the therapeutic void between resection and delayed EBRT while reinforcing treatment of microscopic infiltration beyond the cavity wall.

The therapeutic index of internal radiotherapy critically depends on the physical and biological properties of the radionuclides employed. α-emitters provide exceptional cytotoxicity per track but require stable carriers to prevent off-target effects. β^−^-emitters offer broader tissue coverage but may require fractionation strategies. AE demand subcellular targeting, making them particularly attractive for DNA-directed theranostic platforms. In this context, radionuclide selection emerges as a true therapeutic design parameter. The increasing availability of dual-emission isotopes and recent regulatory authorizations, such as GammaTile^®^ with ¹³¹Cs, further accelerate clinical translation, although rigorous comparative assessments of efficacy and safety across platforms remain lacking. Unlocking its full clinical potential requires more than technological innovation. Its implementation depends on standardized protocols, adaptable technology, and health system readiness. Currently, its clinical evaluation remains limited by heterogeneous inclusion criteria, dosimetric endpoints, and procedural workflows, which hinder cross-study comparison. Harmonization aligned with international guidelines will be essential to generate robust and generalizable evidence.

Technologically, internal radiotherapy stands at the intersection of spatially precise radiation delivery and biologically informed planning. Advances in implantable vectors, injectable platforms, and MC-based simulation tools now enable personalized intraoperative decision-making that extends beyond static dose maps toward dynamically responsive irradiation strategies. This perspective supports the emergence of a biologically guided interventional radiotherapy paradigm, in which patient-specific integrative modeling informs not only dose calculation but also spatial decision-making for source placement. In this framework, multimodal data, including imaging, dosimetry, and tumor biology, can be integrated intraoperatively to identify high-risk regions at the resection margin and guide the spatial distribution of radioactive sources within the surgical cavity. Such an approach moves internal radiotherapy from empirical cavity-based implantation toward a rational, model-driven strategy tailored to the spatial and biological heterogeneity of GBM.

Nevertheless, broader adoption also depends on organizational and economic integration. Many institutions lack nuclear medicine infrastructure, radiopharmacy logistics, or trained personnel. Scalable implementation will require centralized radiopharmaceutical access, remote dosimetric planning capabilities, and interdisciplinary training across neurosurgery, radiation oncology, and nuclear medicine. Reimbursement frameworks also remain insufficiently defined, highlighting the need for early engagement with health systems.

Over the coming years, its integration into GBM management will likely rely on hybrid strategies combining local dose intensification with biologically targeted disruption. Addressing technological, clinical, and organizational barriers is essential to shift this approach from an underused option to a structured component of neuro-oncological care. Taken together, this perspective reframes internal radiotherapy not as an alternative radiation modality, but as a complementary, biologically driven strategy whose clinical impact depends on the development of standardized, integrative dosimetric frameworks. Addressing this gap will be essential for broader clinical adoption in GBM.

## Glossary

AE: Auger electrons
ATM: ataxia-telangiectasia mutated
ATR: ataxia-telangiectasia and rad3-related
AuNPs: gold nanoparticles
BBB: blood-brain barrier
CED: convection-enhanced delivery
CHK2: checkpoint kinase 2
CXCR4: CXC chemokine receptor type 4
DaRT: diffusing alpha-emitters radiation therapy
DNA: deoxyribonucleic acid
DSBs: DNA double-strand breaks
EBRT: external beam RT
ESMO: European Society for Medical Oncology
FAK: focal adhesion kinase
FDA: food and drug administration
GBM: glioblastoma
GSCs: GBM stem-like cells
H2AX: H2A histone family member X
HIF-1α: hypoxia-inducible factor 1 alpha
IL-1β: interleukin-1β
IL-6: interleukin-6
IMRT: intensity-modulated radiotherapy
LET: linear energy transfer
LQ: linear quadratic
L1CAM: L1 cell adhesion molecule
MC: Monte Carlo
MR: magnetic resonance
MRCK: Myotonic dystrophy kinase-related CDC42-binding kinase
MRI: magnetic resonance imaging
NCAM: neural cell adhesion molecule
NCCN: National Comprehensive Cancer Network
NF-κB: nuclear factor-kappa B
NRG: NOD rag2 γc strain
OLIGO2: oligodendrocyte transcription factor 2
PARP: poly(ADP-ribose) polymerase
PDX: patient-derived xenografts
PET: positron emission tomography
ROS: reactive oxygen species
RBE: relative biological effectiveness
RT: radiotherapy
SCID: severe combined immunodeficiency
SPARC: secreted protein acidic and rich in cysteine
SPECT: single-photon emission computed tomography
SOX: SRY-box transcription factor 2
ssDNA: single-stranded DNA
TAT: targeted alpha therapy
TNF-α: tumor necrosis factor alpha
TTFields: tumor-treating fields
VEGF: vascular endothelial growth factor
VMAT: Volumetric Modulated Arc Therapy

## References

[1] Louis DN Perry A Wesseling P Brat DJ Cree IA Figarella-Branger D . The 2021 WHO classification of tumors of the central nervous system: a summary. Neuro-Oncology. (2021) 23:1231–51. doi: 10.1093/neuonc/noab106 34185076 PMC8328013

[2] Molinaro AM Hervey-Jumper S Morshed RA Young J Han SJ Chunduru P . Association of maximal extent of resection of contrast-enhanced and non-contrast-enhanced tumor with survival within molecular subgroups of patients with newly diagnosed glioblastoma. JAMA Oncol. (2020) 6:495–503. doi: 10.1001/jamaoncol.2019.6143 32027343 PMC7042822

[3] Alexander BM Cloughesy TF . Adult glioblastoma. J Clin Oncol. (2017) 35:2402–9. doi: 10.1200/JCO.2017.73.0119 28640706

[4] Stupp R Mason WP van den Bent MJ Weller M Fisher B Taphoorn MJB . Radiotherapy plus concomitant and adjuvant temozolomide for glioblastoma. N Engl J Med. (2005) 352:987–96. doi: 10.1056/NEJMoa043330 15758009

[5] Perry JR Laperriere N O’Callaghan CJ Brandes AA Menten J Phillips C . Short-course radiation plus temozolomide in elderly patients with glioblastoma. N Engl J Med. (2017) 376:1027–37. doi: 10.1056/NEJMoa1611977 28296618

[6] Stupp R Taillibert S Kanner A Read W Steinberg D Lhermitte B . Effect of tumor-treating fields plus maintenance temozolomide vs maintenance temozolomide alone on survival in patients with glioblastoma: a randomized clinical trial. JAMA. (2017) 318:2306–16. doi: 10.1001/jama.2017.18718 29260225 PMC5820703

[7] Fabian D Guillermo Prieto Eibl MP Alnahhas I Sebastian N Giglio P Puduvalli V . Treatment of glioblastoma (GBM) with the addition of tumor-treating fields (TTF): a review. Cancers. (2019) 11:174. doi: 10.3390/cancers11020174 30717372 PMC6406491

[8] McDonald MW Shu H-K Curran WJ Crocker IR . Pattern of failure after limited margin radiotherapy and temozolomide for glioblastoma. Int J Radiat Oncol Biol Phys. (2011) 79:130–6. doi: 10.1016/j.ijrobp.2009.10.048 20399036

[9] Tu Z Xiong H Qiu Y Li G Wang L Peng S . Limited recurrence distance of glioblastoma under modern radiotherapy era. BMC Cancer. (2021) 21:720. doi: 10.1186/s12885-021-08467-3 34154559 PMC8218451

[10] Zheng L Zhou Z-R Yu Q Shi M Yang Y Zhou X . The definition and delineation of the target area of radiotherapy based on the recurrence pattern of glioblastoma after temozolomide chemoradiotherapy. Front Oncol. (2020) 10:615368. doi: 10.3389/fonc.2020.615368 33692942 PMC7937883

[11] Ringel F Pape H Sabel M Krex D Bock HC Misch M . Clinical benefit from resection of recurrent glioblastomas: results of a multicenter study including 503 patients with recurrent glioblastomas undergoing surgical resection. Neuro Oncol. (2016) 18:96–104. doi: 10.1093/neuonc/nov145 26243790 PMC4677413

[12] Bloch O Han SJ Cha S Sun MZ Aghi MK McDermott MW . Impact of extent of resection for recurrent glioblastoma on overall survival: clinical article. J Neurosurg. (2012) 117:1032–8. doi: 10.3171/2012.9.JNS12504 23039151

[13] Shaw E Scott C Souhami L Dinapoli R Kline R Loeffler J . Single dose radiosurgical treatment of recurrent previously irradiated primary brain tumors and brain metastases: final report of RTOG protocol 90-05. Int J Radiat Oncol Biol Phys. (2000) 47:291–8. doi: 10.1016/s0360-3016(99)00507-6 10802351

[14] Fogh SE Andrews DW Glass J Curran W Glass C Champ C . Hypofractionated stereotactic radiation therapy: an effective therapy for recurrent high-grade gliomas. J Clin Oncol. (2010) 28:3048–53. doi: 10.1200/JCO.2009.25.6941 20479391 PMC2982785

[15] Kazmi F Soon YY Leong YH Koh WY Vellayappan B . Re-irradiation for recurrent glioblastoma (GBM): a systematic review and meta-analysis. J Neuro Oncol. (2019) 142:79–90. doi: 10.1007/s11060-018-03064-0 30523605

[16] Tsien CI Pugh SL Dicker AP Raizer JJ Matuszak MM Lallana EC . NRG Oncology/RTOG1205: a randomized phase II trial of concurrent bevacizumab and reirradiation versus bevacizumab alone as treatment for recurrent glioblastoma. J Clin Oncol. (2023) 41:1285–95. doi: 10.1200/JCO.22.00164 36260832 PMC9940937

[17] Wick W Puduvalli VK Chamberlain MC van den Bent MJ Carpentier AF Cher LM . Phase III study of enzastaurin compared with lomustine in the treatment of recurrent intracranial glioblastoma. J Clin Oncol. (2010) 28:1168–74. doi: 10.1200/JCO.2009.23.2595 20124186 PMC2834468

[18] Batchelor TT Mulholland P Neyns B Nabors LB Campone M Wick A . Phase III randomized trial comparing the efficacy of cediranib as monotherapy, and in combination with lomustine, versus lomustine alone in patients with recurrent glioblastoma. J Clin Oncol. (2013) 31:3212–8. doi: 10.1200/JCO.2012.47.2464 23940216 PMC4021043

[19] Jungk C Chatziaslanidou D Ahmadi R Capper D Bermejo JL Exner J . Chemotherapy with BCNU in recurrent glioma: analysis of clinical outcome and side effects in chemotherapy-naïve patients. BMC Cancer. (2016) 16:81. doi: 10.1186/s12885-016-2131-6 26865253 PMC4748520

[20] Garcia CR Slone SA Morgan RM Gruber L Kumar SS Lightner DD . Dose-dense temozolomide for recurrent high-grade gliomas: a single-center retrospective study. Med Oncol. (2018) 35:136. doi: 10.1007/s12032-018-1198-0 30155806

[21] Roussakow SV . Clinical and economic evaluation of modulated electrohyperthermia concurrent to dose-dense temozolomide 21/28 days regimen in the treatment of recurrent glioblastoma: a retrospective analysis of a two-centre German cohort trial with systematic comparison and effect-to-treatment analysis. BMJ Open. (2017) 7:e017387. doi: 10.1136/bmjopen-2017-017387 29102988 PMC5722101

[22] Cloughesy T Finocchiaro G Belda-Iniesta C Recht L Brandes AA Pineda E . Randomized, double-blind, placebo-controlled, multicenter phase II study of onartuzumab plus bevacizumab versus placebo plus bevacizumab in patients with recurrent glioblastoma: efficacy, safety, and hepatocyte growth factor and O6-methylguanine-DNA methyltransferase biomarker analyses. J Clin Oncol. (2017) 35:343–51. doi: 10.1200/JCO.2015.64.7685 27918718

[23] Reardon DA Brandes AA Omuro A Mulholland P Lim M Wick A . Effect of nivolumab vs bevacizumab in patients with recurrent glioblastoma: the CheckMate 143 phase 3 randomized clinical trial. JAMA Oncol. (2020) 6:1003–10. doi: 10.1001/jamaoncol.2020.1024 32437507 PMC7243167

[24] Cloughesy TF Mochizuki AY Orpilla JR Hugo W Lee AH Davidson TB . Neoadjuvant anti-PD-1 immunotherapy promotes a survival benefit with intratumoral and systemic immune responses in recurrent glioblastoma. Nat Med. (2019) 25:477–86. doi: 10.1038/s41591-018-0337-7 30742122 PMC6408961

[25] Ferguson SD Zhou S Huse JT de Groot JF Xiu J Subramaniam DS . Targetable gene fusions associate with the IDH wild-type astrocytic lineage in adult gliomas. J Neuropathol Exp Neurol. (2018) 77:437–42. doi: 10.1093/jnen/nly022 29718398 PMC5961205

[26] El Atat O Naser R Abdelkhalek M Habib RA El Sibai M . Molecular targeted therapy: a new avenue in glioblastoma treatment. Oncol Lett. (2023) 25:46. doi: 10.3892/ol.2022.13632 36644133 PMC9811647

[27] D’Alessio A Proietti G Sica G Scicchitano BM . Pathological and molecular features of glioblastoma and its peritumoral tissue. Cancers (Basel). (2019) 11:469. doi: 10.3390/cancers11040469 30987226 PMC6521241

[28] Romanelli P Conti A Pontoriero A Ricciardi GK Tomasello F De Renzis C . Role of stereotactic radiosurgery and fractionated stereotactic radiotherapy for the treatment of recurrent glioblastoma multiforme. Neurosurg Focus. (2009) 27:E8. doi: 10.3171/2009.9.FOCUS09187 19951061

[29] Sharma M Schroeder JL Elson P Meola A Barnett GH Vogelbaum MA . Outcomes and prognostic stratification of patients with recurrent glioblastoma treated with salvage stereotactic radiosurgery. J Neurosurg. (2019) 131:489–99. doi: 10.3171/2018.4.JNS172909 30485180

[30] Imber BS Kanungo I Braunstein S Barani IJ Fogh SE Nakamura JL . Indications and efficacy of Gamma Knife stereotactic radiosurgery for recurrent glioblastoma: 2 decades of institutional experience. Neurosurgery. (2017) 80:129–39. doi: 10.1227/NEU.0000000000001344 27428784 PMC5235998

[31] Bunevicius A Sheehan JP . Radiosurgery for glioblastoma. Neurosurg Clinics North America. (2021) 32:117–28. doi: 10.1016/j.nec.2020.08.007 33223020

[32] Saeed AM Khairnar R Sharma AM Larson GL Tsai HK Wang CJ . Clinical outcomes in patients with recurrent glioblastoma treated with proton beam therapy reirradiation: analysis of the multi-institutional Proton Collaborative Group Registry. Adv Radiat Oncol. (2020) 5:978–83. doi: 10.1016/j.adro.2020.03.022 33083661 PMC7557126

[33] Pellettieri L H-Stenstam B Rezaei A Giusti V Sköld K . An investigation of boron neutron capture therapy for recurrent glioblastoma multiforme. Acta Neurol Scand. (2008) 117:191–7. doi: 10.1111/j.1600-0404.2007.00924.x 18297764

[34] Shimizu S Nakai K Li Y Mizumoto M Kumada H Ishikawa E . Boron neutron capture therapy for recurrent glioblastoma multiforme: imaging evaluation of a case with long-term local control and survival. Cureus. (2023) 15:e33898. doi: 10.7759/cureus.33898 36819302 PMC9937644

[35] De Barros A Attal J Roques M Nicolau J Sol J-C Cohen-Jonathan-Moyal E . Impact on survival of early tumor growth between surgery and radiotherapy in patients with de novo glioblastoma. J Neuro Oncol. (2019) 142:489–97. doi: 10.1007/s11060-019-03120-3 30783874

[36] Stummer W Pichlmeier U Meinel T Wiestler OD Zanella F Reulen H-J . Fluorescence-guided surgery with 5-aminolevulinic acid for resection of Malignant glioma: a randomised controlled multicentre phase III trial. Lancet Oncol. (2006) 7:392–401. doi: 10.1016/S1470-2045(06)70665-9 16648043

[37] Petrecca K Guiot M-C Panet-Raymond V Souhami L . Failure pattern following complete resection plus radiotherapy and temozolomide is at the resection margin in patients with glioblastoma. J Neuro Oncol. (2013) 111:19–23. doi: 10.1007/s11060-012-0983-4 23054563

[38] Behbahaninia M Martirosyan NL Georges J Udovich JA Kalani MYS Feuerstein BG . Intraoperative fluorescent imaging of intracranial tumors: a review. Clin Neurol Neurosurg. (2013) 115:517–28. doi: 10.1016/j.clineuro.2013.02.019 23523009

[39] Bao S Wu Q McLendon RE Hao Y Shi Q Hjelmeland AB . Glioma stem cells promote radioresistance by preferential activation of the DNA damage response. Nature. (2006) 444:756–60. doi: 10.1038/nature05236 17051156

[40] Zhong L Chen L Lv S Li Q Chen G Luo W . Efficacy of moderately hypofractionated simultaneous integrated boost intensity-modulated radiotherapy combined with temozolomide for the postoperative treatment of glioblastoma multiforme: a single-institution experience. Radiat Oncol. (2019) 14:104. doi: 10.1186/s13014-019-1305-1 31196126 PMC6567425

[41] Ylanan AMD Pascual JSG Cruz-Lim EMD Ignacio KHD Cañal JPA Khu KJO . Intraoperative radiotherapy for glioblastoma: a systematic review of techniques and outcomes. J Clin Neurosci. (2021) 93:36–41. doi: 10.1016/j.jocn.2021.08.022 34656258

[42] D’Amico RS Aghi MK Vogelbaum MA Bruce JN . Convection-enhanced drug delivery for glioblastoma: a review. J Neuro Oncol. (2021) 151:415–27. doi: 10.1007/s11060-020-03408-9 33611708 PMC8034832

[43] Ellingson BM Nguyen HN Lai A Nechifor RE Zaw O Pope WB . Contrast-enhancing tumor growth dynamics of preoperative, treatment-naive human glioblastoma. Cancer. (2016) 122:1718–27. doi: 10.1002/cncr.29957 26998740

[44] Schulz JA Rodgers LT Kryscio RJ Hartz AMS Bauer B . Characterization and comparison of human glioblastoma models. BMC Cancer. (2022) 22:844. doi: 10.1186/s12885-022-09910-9 35922758 PMC9347152

[45] Zhang M Xu F Ni W Qi W Cao W Xu C . Survival impact of delaying postoperative chemoradiotherapy in newly-diagnosed glioblastoma patients. Trans Cancer Res. (2020) 9(9):5450–8. doi: 10.21037/tcr-20-1718 35117910 PMC8797492

[46] Nordal RA Wong CS . Molecular targets in radiation-induced blood-brain barrier disruption. Int J Radiat Oncol Biol Phys. (2005) 62:279–87. doi: 10.1016/j.ijrobp.2005.01.039 15850934

[47] Greene-Schloesser D Robbins ME Peiffer AM Shaw EG Wheeler KT Chan MD . Radiation-induced brain injury: a review. Front Oncol. (2012) 2:73. doi: 10.3389/fonc.2012.00073 22833841 PMC3400082

[48] Fauquette W Amourette C Dehouck M-P Diserbo M . Radiation-induced blood-brain barrier damages: an *in vitro* study. Brain Res. (2012) 1433:114–26. doi: 10.1016/j.brainres.2011.11.022 22153623

[49] Huang R-X Zhou P-K . DNA damage response signaling pathways and targets for radiotherapy sensitization in cancer. Sig Transduct Target Ther. (2020) 5:60. doi: 10.1038/s41392-020-0150-x 32355263 PMC7192953

[50] Wu Y Song Y Wang R Wang T . Molecular mechanisms of tumor resistance to radiotherapy. Mol Cancer. (2023) 22:96. doi: 10.1186/s12943-023-01801-2 37322433 PMC10268375

[51] Yan S Sorrell M Berman Z . Functional interplay between ATM/ATR-mediated DNA damage response and DNA repair pathways in oxidative stress. Cell Mol Life Sci. (2014) 71:3951–67. doi: 10.1007/s00018-014-1666-4 24947324 PMC4176976

[52] Vellayappan B Tan CL Yong C Khor LK Koh WY Yeo TT . Diagnosis and management of radiation necrosis in patients with brain metastases. Front Oncol. (2018) 8:395. doi: 10.3389/fonc.2018.00395 30324090 PMC6172328

[53] Lumniczky K Szatmári T Sáfrány G . Ionizing radiation-induced immune and inflammatory reactions in the brain. Front Immunol. (2017) 8:517. doi: 10.3389/fimmu.2017.00517 28529513 PMC5418235

[54] Nonoguchi N Miyatake S-I Fukumoto M Furuse M Hiramatsu R Kawabata S . The distribution of vascular endothelial growth factor-producing cells in clinical radiation necrosis of the brain: pathological consideration of their potential roles. J Neuro Oncol. (2011) 105:423–31. doi: 10.1007/s11060-011-0610-9 21688077

[55] Greene-Schloesser D Robbins ME . Radiation-induced cognitive impairment--from bench to bedside. Neuro Oncol. (2012) 14 Suppl 4:iv37–44. doi: 10.1093/neuonc/nos196 23095829 PMC3480242

[56] Chapman JD . Can the two mechanisms of tumor cell killing by radiation be exploited for therapeutic gain? J Radiat Res. (2014) 55:2–9. doi: 10.1093/jrr/rrt111 24105710 PMC3885134

[57] Badiyan SN Markovina S Simpson JR Robinson CG DeWees T Tran DD . Radiation therapy dose escalation for glioblastoma multiforme in the era of temozolomide. Int J Radiat Oncol Biol Phys. (2014) 90:877–85. doi: 10.1016/j.ijrobp.2014.07.014 25257812

[58] Prados MD Wara WM Sneed PK McDermott M Chang SM Rabbitt J . Phase III trial of accelerated hyperfractionation with or without difluromethylornithine (DFMO) versus standard fractionated radiotherapy with or without DFMO for newly diagnosed patients with glioblastoma multiforme. Int J Radiat Oncol Biol Phys. (2001) 49:71–7. doi: 10.1016/s0360-3016(00)01458-9 11163499

[59] Roa W Brasher PMA Bauman G Anthes M Bruera E Chan A . Abbreviated course of radiation therapy in older patients with glioblastoma multiforme: a prospective randomized clinical trial. J Clin Oncol. (2004) 22:1583–8. doi: 10.1200/JCO.2004.06.082 15051755

[60] Heddleston JM Li Z McLendon RE Hjelmeland AB Rich JN . The hypoxic microenvironment maintains glioblastoma stem cells and promotes reprogramming towards a cancer stem cell phenotype. Cell Cycle. (2009) 8:3274–84. doi: 10.4161/cc.8.20.9701 19770585 PMC2825672

[61] Guerrero PA Tchaicha JH Chen Z Morales JE McCarty N Wang Q . Glioblastoma stem cells exploit the αvβ8 integrin-TGFβ1 signaling axis to drive tumor initiation and progression. Oncogene. (2017) 36:6568–80. doi: 10.1038/onc.2017.248 28783169 PMC5882487

[62] Gu J Mu N Jia B Guo Q Pan L Zhu M . Targeting radiation-tolerant persister cells as a strategy for inhibiting radioresistance and recurrence in glioblastoma. Neuro-Oncology. (2022) 24:1056–70. doi: 10.1093/neuonc/noab288 34905060 PMC9248405

[63] Wu H Guo C Wang C Xu J Zheng S Duan J . Single-cell RNA sequencing reveals tumor heterogeneity, microenvironment, and drug-resistance mechanisms of recurrent glioblastoma. Cancer Sci. (2023) 114:2609–21. doi: 10.1111/cas.15773 36853018 PMC10236634

[64] Alves ALV Gomes INF Carloni AC Rosa MN da Silva LS Evangelista AF . Role of glioblastoma stem cells in cancer therapeutic resistance: a perspective on antineoplastic agents from natural sources and chemical derivatives. Stem Cell Res Ther. (2021) 12:206. doi: 10.1186/s13287-021-02231-x 33762015 PMC7992331

[65] Che P Yu L Friedman GK Wang M Ke X Wang H . Integrin αvβ3 engagement regulates glucose metabolism and migration through focal adhesion kinase (FAK) and protein arginine methyltransferase 5 (PRMT5) in glioblastoma cells. Cancers. (2021) 13:1111. doi: 10.3390/cancers13051111 33807786 PMC7961489

[66] Pace KR Dutt R Galileo DS . Exosomal L1CAM stimulates glioblastoma cell motility, proliferation, and invasiveness. Int J Mol Sci. (2019) 20:3982. doi: 10.3390/ijms20163982 31426278 PMC6720723

[67] Wirthschaft P Bode J Soni H Dietrich F Krüwel T Fischer B . RhoA regulates translation of the Nogo-A decoy SPARC in white matter-invading glioblastomas. Acta Neuropathol. (2019) 138:275–93. doi: 10.1007/s00401-019-02021-z 31062076 PMC6660512

[68] Hambardzumyan D Gutmann DH Kettenmann H . The role of microglia and macrophages in glioma maintenance and progression. Nat Neurosci. (2016) 19:20–7. doi: 10.1038/nn.4185 26713745 PMC4876023

[69] Knudsen AM Halle B Cédile O Burton M Baun C Thisgaard H . Surgical resection of glioblastomas induces pleiotrophin-mediated self-renewal of glioblastoma stem cells in recurrent tumors. Neuro-Oncology. (2022) 24:1074–87. doi: 10.1093/neuonc/noab302 34964899 PMC9248408

[70] Venkataramani V Yang Y Schubert MC Reyhan E Tetzlaff SK Wißmann N . Glioblastoma hijacks neuronal mechanisms for brain invasion. Cell. (2022) 185:2899–2917.e31. doi: 10.1016/j.cell.2022.06.054 35914528

[71] Venkatesh HS Johung TB Caretti V Noll A Tang Y Nagaraja S . Neuronal activity promotes glioma growth through neuroligin-3 secretion. Cell. (2015) 161:803–16. doi: 10.1016/j.cell.2015.04.012 25913192 PMC4447122

[72] Birch JL Gilmore LD Strathdee K McKinnon H Drysdale M Olson M . P08.05 irradiation of glioblastoma cells can promote enhanced motility and invasiveness, both *in vitro* and *in vivo* through activation of MRCK. Neuro-Oncology. (2017) 19:iii54. doi: 10.1093/neuonc/nox036.195

[73] Smith K Nakaji P Thomas T Pinnaduwage D Wallstrom G Choi M . Safety and patterns of survivorship in recurrent GBM following resection and surgically targeted radiation therapy: results from a prospective trial. Neuro Oncol. (2022) 24:S4–S15. doi: 10.1093/neuonc/noac133 36322102 PMC9629483

[74] Gessler DJ Neil EC Shah R Levine J Shanks J Wilke C . GammaTile® brachytherapy in the treatment of recurrent glioblastomas. Neuro Oncol Adv. (2022) 4:vdab185. doi: 10.1093/noajnl/vdab185 35088050 PMC8788013

[75] Chan TA Weingart JD Parisi M Hughes MA Olivi A Borzillary S . Treatment of recurrent glioblastoma multiforme with GliaSite brachytherapy. Int J Radiat Oncol Biol Phys. (2005) 62:1133–9. doi: 10.1016/j.ijrobp.2004.12.032 15990019

[76] Chino K Silvain D Grace A Stubbs J Stea B . Feasibility and safety of outpatient brachytherapy in 37 patients with brain tumors using the GliaSite® radiation therapy system: outpatient brachytherapy for brain tumors using gliasite balloon. Med Phys. (2008) 35:3383–8. doi: 10.1118/1.2940602 18697562

[77] Waters JD Rose B Gonda DD Scanderbeg DJ Russell M Alksne JF . Immediate post-operative brachytherapy prior to irradiation and temozolomide for newly diagnosed glioblastoma. J Neuro Oncol. (2013) 113:467–77. doi: 10.1007/s11060-013-1139-x 23673513

[78] Tanderup K Ménard C Polgar C Lindegaard JC Kirisits C Pötter R . Advancements in brachytherapy. Adv Drug Delivery Rev. (2017) 109:15–25. doi: 10.1016/j.addr.2016.09.002 27637454

[79] Bolcaen J Kleynhans J Nair S Verhoeven J Goethals I Sathekge M . A perspective on the radiopharmaceutical requirements for imaging and therapy of glioblastoma. Theranostics. (2021) 11:7911–47. doi: 10.7150/thno.56639 34335972 PMC8315062

[80] Simon J-M Cornu P Boisserie G Hasboun D Tep B Hardiman C . Brachytherapy of glioblastoma recurring in previously irradiated territory: predictive value of tumor volume. Int J Radiat Oncol Biol Phys. (2002) 53:67–74. doi: 10.1016/S0360-3016(01)02804-8 12007943

[81] Bentsion DL Gvozdev PB Sakovich VP Fialko NV Kolotvinov VS Baiankina SN . The first experience in interstitial brachytherapy for primary and metastatic tumors of the brain. Zh Vopr Neirokhir Im N N Burdenko. (2006), 18–21. discussion 21. 16739930

[82] He X Liu M Zhang M Sequeiros RB Xu Y Wang L . A novel three-dimensional template combined with MR-guided 125I brachytherapy for recurrent glioblastoma. Radiat Oncol. (2020) 15:146. doi: 10.1186/s13014-020-01586-4 32513276 PMC7282063

[83] Lee KK Lee JY Nam JM Kim CB Park KR . High-dose-rate vs. low-dose-rate intracavitary brachytherapy for carcinoma of the uterine cervix: systematic review and meta-analysis. Brachytherapy. (2015) 14:449–57. doi: 10.1016/j.brachy.2015.02.390 25906951

[84] Choe S-I Lee Y Habashi R Samarasinghe Y Lee MH McKechnie T . The role of brachytherapy in treatment of stage I esophageal cancer: a systematic review. Brachytherapy. (2022) 21:877–86. doi: 10.1016/j.brachy.2022.05.007 35941072

[85] Campos L Stabin M . Intravascular brachytherapy to prevent restenosis: dosimetric considerations. Cell Mol Biol (Noisy-le-grand). (2002) 48:429–39. 12146694

[86] Skowronek J . Brachytherapy in the treatment of skin cancer: an overview. Adv Dermatol Allergol. (2015) 32:362–7. doi: 10.5114/pdia.2015.54746 26759545 PMC4692821

[87] Wahl RL Sgouros G Iravani A Jacene H Pryma D Saboury B . Normal-tissue tolerance to radiopharmaceutical therapies, the knowns and the unknowns. J Nucl Med. (2021) 62:23S–35S. doi: 10.2967/jnumed.121.262751 34857619 PMC12079726

[88] Dale RG Coles IP Deehan C O’Donoghue JA . Calculation of integrated biological response in brachytherapy. Int J Radiat Oncol Biol Phys. (1997) 38:633–42. doi: 10.1016/s0360-3016(97)00096-5 9231690

[89] Mare SD Nishri Y Shai A Efrati M Deutsch L Den RB . Diffusing alpha-emitters radiation therapy promotes a proimmunogenic tumor microenvironment and synergizes with programmed cell death protein 1 blockade. Int J Radiat Oncol Biol Phys. (2023) 115:707–18. doi: 10.1016/j.ijrobp.2022.08.043 36031029

[90] Nishri Y Vatarescu M Luz I Epstein L Dumančić M Del Mare S . Diffusing alpha-emitters radiation therapy in combination with temozolomide or bevacizumab in human glioblastoma multiforme xenografts. Front Oncol. (2022) 12:888100. doi: 10.3389/fonc.2022.888100 36237307 PMC9552201

[91] Shoshan Y Gomori MJ Moss L Bari SE Edery N Den RB . Stereotactic implantation of diffusing alpha-emitters radiation therapy sources in the swine brain: a potential new focal therapy for brain tumors. J Neuro Oncol. (2025) 172:387–96. doi: 10.1007/s11060-024-04919-5 39747715 PMC11937107

[92] Poty S Francesconi LC McDevitt MR Morris MJ Lewis JS . α-Emitters for radiotherapy: from basic radiochemistry to clinical studies—part 1. J Nucl Med. (2018) 59:878–84. doi: 10.2967/jnumed.116.186338 29545378 PMC6004557

[93] Sattiraju A Solingapuram Sai KK Xuan A Pandya DN Almaguel FG Wadas TJ . IL13RA2 targeted alpha particle therapy against glioblastomas. Oncotarget. (2017) 8:42997–3007. doi: 10.18632/oncotarget.17792 28562337 PMC5522122

[94] Krolicki L Bruchertseifer F Kunikowska J Koziara H Królicki B Jakuciński M . Prolonged survival in secondary glioblastoma following local injection of targeted alpha therapy with 213Bi-substance P analogue. Eur J Nucl Med Mol Imaging. (2018) 45:1636–44. doi: 10.1007/s00259-018-4015-2 29713762 PMC6061489

[95] Roncali L Marionneau-Lambot S Roy C Eychenne R Gouard S Avril S . Brain intratumoural astatine-211 radiotherapy targeting syndecan-1 leads to durable glioblastoma remission and immune memory in female mice. EBioMedicine. (2024) 105:105202. doi: 10.1016/j.ebiom.2024.105202 38905749 PMC11246004

[96] Ackerman NL de la Fuente Rosales L Falzone N Vallis KA Bernal MA . Targeted alpha therapy with 212Pb or 225Ac: change in RBE from daughter migration. Physica Med. (2018) 51:91–8. doi: 10.1016/j.ejmp.2018.05.020 29807854

[97] Ku A Facca VJ Cai Z Reilly RM . Auger electrons for cancer therapy – a review. EJNMMI Radiopharm Chem. (2019) 4:27. doi: 10.1186/s41181-019-0075-2 31659527 PMC6800417

[98] Pirovano G Jannetti SA Carter LM Sadique A Kossatz S Guru N . Targeted brain tumor radiotherapy using an Auger emitter. Clin Cancer Res. (2020) 26:2871–81. doi: 10.1158/1078-0432.CCR-19-2440 32066626 PMC7299758

[99] Thisgaard H Halle B Aaberg-Jessen C Olsen BB Therkelsen ASN Dam JH . Highly effective Auger-electron therapy in an orthotopic glioblastoma xenograft model using convection-enhanced delivery. Theranostics. (2016) 6:2278–91. doi: 10.7150/thno.15898 27924163 PMC5135448

[100] Khazaei Monfared Y Heidari P Klempner SJ Mahmood U Parikh AR Hong TS . DNA damage by radiopharmaceuticals and mechanisms of cellular repair. Pharmaceutics. (2023) 15:2761. doi: 10.3390/pharmaceutics15122761 38140100 PMC10748326

[101] Elgqvist J Frost S Pouget J-P Albertsson P . The potential and hurdles of targeted alpha therapy – clinical trials and beyond. Front Oncol. (2014) 3. doi: 10.3389/fonc.2013.00324 24459634 PMC3890691

[102] Pouget J-P Lozza C Deshayes E Boudousq V Navarro-Teulon I . Introduction to radiobiology of targeted radionuclide therapy. Front Med. (2015) 2. doi: 10.3389/fmed.2015.00012 25853132 PMC4362338

[103] Pouget J-P Constanzo J . Revisiting the radiobiology of targeted alpha therapy. Front Med (Lausanne). (2021) 8:692436. doi: 10.3389/fmed.2021.692436 34386508 PMC8353448

[104] Allard E Hindre F Passirani C Lemaire L Lepareur N Noiret N . 188Re-loaded lipid nanocapsules as a promising radiopharmaceutical carrier for internal radiotherapy of Malignant gliomas. Eur J Nucl Med Mol Imaging. (2008) 35:1838–46. doi: 10.1007/s00259-008-0735-z 18465130 PMC2737004

[105] Séhédic D Chourpa I Tétaud C Griveau A Loussouarn C Avril S . Locoregional confinement and major clinical benefit of 188Re-loaded CXCR4-targeted nanocarriers in an orthotopic human to mouse model of glioblastoma. Theranostics. (2017) 7:4517–36. doi: 10.7150/thno.19403 29158842 PMC5695146

[106] Georgiou CJ Cai Z Alsaden N Cho H Behboudi M Winnik MA . Treatment of orthotopic U251 human glioblastoma multiforme tumors in NRG mice by convection-enhanced delivery of gold nanoparticles labeled with the β-particle-emitting radionuclide, 177Lu. Mol Pharm. (2023) 20:582–92. doi: 10.1021/acs.molpharmaceut.2c00815 36516432 PMC9812026

[107] Boros E Packard AB . Radioactive transition metals for imaging and therapy. Chem Rev. (2019) 119:870–901. doi: 10.1021/acs.chemrev.8b00281 30299088

[108] Tamihardja J Weick S Lutyj P Zimmermann M Bratengeier K Flentje M . Comparing iridium-192 with cobalt-60 sources in high-dose-rate brachytherapy boost for localized prostate cancer. Acta Oncol. (2022) 61:714–9. doi: 10.1080/0284186X.2022.2068968 35485446

[109] Sharma DN Kumar P Subramani V Giridhar P . Low-dose-rate, high-dose-rate, and pulsed-dose-rate intra-cavitary brachytherapy for cervical cancer: The very first comparison study. Jcb. (2024) 16:273–8. doi: 10.5114/jcb.2024.142938 39628820 PMC11609853

[110] Yogo K Misawa M Shimizu M Shimizu H Kitagawa T Hirayama R . Effect of gold nanoparticle radiosensitization on plasmid DNA damage induced by high-dose-rate brachytherapy. IJN. (2021) 16:359–70. doi: 10.2147/IJN.S292105 33469290 PMC7813456

[111] Tatter SB Shaw EG Rosenblum ML Karvelis KC Kleinberg L Weingart J . An inflatable balloon catheter and liquid 125I radiation source (GliaSite Radiation Therapy System) for treatment of recurrent Malignant glioma: multicenter safety and feasibility trial. J Neurosurg. (2003) 99:297–303. doi: 10.3171/jns.2003.99.2.0297 12924704

[112] Huh R Park YS Lee JD Chung YS Park YG Chung SS . Therapeutic effects of holmium-166 chitosan complex in rat brain tumor model. Yonsei Med J. (2005) 46:51. doi: 10.3349/ymj.2005.46.1.51 15744805 PMC2823057

[113] Francis Z Incerti S Capra R Mascialino B Montarou G Stepan V . Molecular scale track structure simulations in liquid water using the Geant4-DNA Monte-Carlo processes. Appl Radiat Isot. (2011) 69:220–6. doi: 10.1016/j.apradiso.2010.08.011 20810287

[114] Alcocer-Ávila M Levrague V Delorme R Testa É Beuve M . Biophysical modeling of low-energy ion irradiations with NanOx. Med Phys. (2024) 51:9358–71. doi: 10.1002/mp.17407 39287463

[115] Lampe N Karamitros M Breton V Brown JMC Kyriakou I Sakata D . Mechanistic DNA damage simulations in Geant4-DNA part 1: A parameter study in a simplified geometry. Phys Med. (2018) 48:135–45. doi: 10.1016/j.ejmp.2018.02.011 29628360

[116] Lampe N Karamitros M Breton V Brown JMC Sakata D Sarramia D . Mechanistic DNA damage simulations in Geant4-DNA part 2: Electron and proton damage in a bacterial cell. Phys Med. (2018) 48:146–55. doi: 10.1016/j.ejmp.2017.12.008 29371062

[117] Fois GR Tran HN Fiegel V Blain G Chiavassa S Craff E . Monte Carlo simulations of microdosimetry and radiolytic species production at long time post proton irradiation using GATE and Geant4-DNA. Med Phys. (2024) 51:7500–10. doi: 10.1002/mp.17281 38976841

[118] Sabri ME Moghaddasi L Wilson P Saran F Bezak E . Targeted alpha therapy for glioblastoma: Review on *in vitro*, *in vivo* and clinical trials. Target Oncol. (2024) 19:511–31. doi: 10.1007/s11523-024-01071-y 38836953 PMC11230998

[119] Prasad V . Beyond becquerel and biology to precision radiomolecular oncology: festschrift in honor of Richard P. Baum. In: Springer International Publishing. Cham: Springer International Publishing (2024). doi: 10.1007/978-3-031-33533-4

[120] Ekhator C Nwankwo I Rak E Homayoonfar A Fonkem E Rak R . GammaTile: Comprehensive review of a novel radioactive intraoperative seed-loading device for the treatment of brain tumors. Cureus. (2022) 14:e29970. doi: 10.7759/cureus.29970 36225241 PMC9541893

[121] Seidel S Garvalov BK Wirta V von Stechow L Schänzer A Meletis K . A hypoxic niche regulates glioblastoma stem cells through hypoxia inducible factor 2 alpha. Brain. (2010) 133:983–95. doi: 10.1093/brain/awq042 20375133

[122] Li Z Bao S Wu Q Wang H Eyler C Sathornsumetee S . Hypoxia-inducible factors regulate tumorigenic capacity of glioma stem cells. Cancer Cell. (2009) 15:501–13. doi: 10.1016/j.ccr.2009.03.018 19477429 PMC2693960

[123] Xie XP Laks DR Sun D Ganbold M Wang Z Pedraza AM . Quiescent human glioblastoma cancer stem cells drive tumor initiation, expansion, and recurrence following chemotherapy. Dev Cell. (2022) 57:32–46.e8. doi: 10.1016/j.devcel.2021.12.007 35016005 PMC8820651

